# Supplementary material for: Interdisciplinary interventions that improve patient-reported outcomes in perioperative cancer care: A systematic review of randomized control trials
Source: PLoS One. 2023 Nov 20;18(11):e0294599. doi: 10.1371/journal.pone.0294599 (PMC10659207; doi:10.1371/journal.pone.0294599)
Supplement: S1 File — (DOCX) [file pone.0294599.s002.docx]

**Appendix S1:** Search Strategy

PubMed (06/10/2021) - 3461; (03/30/2023) - 659

("physician assistants"[Mesh] OR "physician assistant*"[tw] OR "advanced-practi*"[tw] OR "advance-practi*"[tw] OR "advanced practitioner*"[tw] OR "advance practice nurse*" OR "advanced practice nurse*" OR "advance practice provider*" OR "advanced practice provider*" OR "advanced care provider*" OR "nurse practitioners"[Mesh] OR "nurse specialists"[Mesh] OR "nurse clinicians"[Mesh] OR “perioperative nurse*”[tw] OR "social worker*"[tw] OR "social workers"[Mesh] OR “perioperative nursing”[Mesh] OR nurses[Mesh] OR “nurses”[tw] OR “nurse”[tw] OR “patient care team”[Mesh] OR ((interdisciplinary[tw] OR multidisciplinary[tw] OR palliative[tw]) AND (healthcare[tiab] OR care[tiab]) AND (“team”[tiab] OR “teams”[tiab])) ) AND (cancer*[tw] OR neoplas*[tw] OR carcinoma*[tw] OR malignan*[tw] OR tumor*[tw] OR oncolog*[tw] OR sarcoma*[tw] OR adenocarcinoma[tw] OR metasta*[tw] OR neoplasms[Mesh] OR tumour[tw] OR tumours[tw] OR leukemia*[tw] OR lymphoma[tw] OR melanoma[tw] OR blastoma[tw]) AND ("randomized controlled trial"[PT] OR "controlled clinical trial"[PT] OR "random*"[tiab] OR "placebo"[tiab] OR "clinical trials as topic"[MH] OR "randomly"[tiab] OR "trial"[ti] OR "clinical trial, phase III"[pt] OR "phase 3"[tw] OR "phase3"[tw] OR "phase III"[TW] OR "random allocation"[MH] OR "double-blind method"[MH] OR "single-blind method"[MH] OR "clinical trial"[pt] OR "clinical trial, phase I"[pt] OR "clinical trial, phase II"[pt] OR "clinical trial, phase IV"[pt] OR "multicenter study"[PT] OR "clinical trial*"[tiab] OR placebo*[tiab] OR sham[tiab] OR “single blind*”[tw] OR “double blind*”[tw]) NOT ((review[PT] OR “observational study”[PT] OR "Observational Study, Veterinary”[PT] OR “clinical trial, veterinary”[PT] OR “comparative study”[PT]) NOT “clinical trial”[PT])

Embase (06.18.2021) - 4201; (03/30/2023) - 726

("physician assistant"/exp OR "physician assistant*":ti,ab,kw OR “advanced practice provider”/exp OR ((advanced OR advance) Next/2 practi*)  OR “advanced practice nurse”/exp OR "nurse practitioner"/exp OR “nurse practitioner*”:ti,ab,kw OR "nurse specialist"/exp OR “nurse specialist*”:ti,ab,kw OR "nurse clinician*":ti,ab,kw OR “clinical nurse specialist”/exp OR “perioperative nurse*”:ti,ab,kw OR "social worker*":ti,ab,kw OR "social worker"/exp OR “perioperative nursing”/exp OR nurse/exp OR “nurses”:ti,ab,kw OR “nurse”:ti,ab,kw OR “collaborative care team”/exp OR “patient care team*”:ti,ab,kw OR ((interdisciplinary OR multidisciplinary OR palliative OR collaborative) Next/4 (team*)) ) AND (cancer*:ti,ab,kw OR neoplas*:ti,ab,kw OR carcinoma*:ti,ab,kw OR malignan*:ti,ab,kw OR tumor*:ti,ab,kw OR oncolog*:ti,ab,kw OR sarcoma*:ti,ab,kw OR adenocarcinoma:ti,ab,kw OR metasta*:ti,ab,kw OR “malignant neoplasm”/exp OR tumour:ti,ab,kw OR tumours:ti,ab,kw OR leukemia*:ti,ab,kw OR lymphoma:ti,ab,kw OR melanoma:ti,ab,kw OR blastoma:ti,ab,kw) AND ("randomized controlled trial"/de OR "controlled clinical trial"/exp OR "random*":ti,ab OR "placebo":ti,ab OR "trial":ti OR "single blind procedure"/de OR "double blind procedure"/de OR "phase 4 clinical trial"/de OR “phase 3 clinical trial”/de OR “phase 2 clinical trial“/de) NOT (“review”/de )   NOT ([animals]/lim NOT [humans]/lim)

CINAHL  (7.6.21) - 2048; (03/30/2023) - 127

(MH "physician assistant" OR "physician assistant*" OR MH “advanced practice nurses” OR ((advanced OR advance) W2 practi*)  OR MH "nurse practitioners" OR “nurse practitioner*” OR MH "clinical nurse specialists" OR “nurse specialist*” OR "nurse clinician*" OR “perioperative nurse*” OR “surgical nurse*” OR "social worker*" OR MH "social workers" OR MH “perioperative nursing” OR MH “nurses” OR “nurses” OR “nurse” OR MH “multidisciplinary care team” OR “collaborative care team*” OR “patient care team*” OR ((interdisciplinary OR multidisciplinary OR palliative OR collaborative) W4 (team*)) ) AND (cancer* OR neoplas* OR carcinoma* OR malignan* OR tumor* OR oncolog* OR sarcoma* OR adenocarcinoma OR metasta* OR MH“neoplasms” OR tumour OR tumours OR leukemia* OR lymphoma OR melanoma OR blastoma) AND (MH "randomized controlled trials" OR "controlled clinical trial*" OR TI "random*” OR AB “random*” OR TI "placebo" OR AB “placebo” OR TI "trial" OR MH "single-blind studies" OR MH "double-blind studies" OR MH “triple-blind studies” OR "phase 4 clinical trial*" OR “phase 3 clinical trial*”  OR “phase 2 clinical trial*“) NOT (MH “literature review” )   NOT (MH “animals” NOT MH “human”)

**Appendix S2:** Abstraction Guide

**Identification**

**Study Details**

Sponsorship source: *The source of funding for this project.*

Country:

Setting:

Publication date:

Comments:

**Author’s contact details**

Author’s name:

Institution:

Email:

Address:

**Methods**

Design:

**Population**

Inclusion Criteria: *Write out the study’s inclusion criteria for participants*

Exclusion Criteria: *Write out the study’s exclusion criteria for participants*

Group Differences: *If no differences, type “N/A” (because the studies should be randomized controlled trials, there likely will be no significant difference within the groups that impact the analysis of the outcomes).*

**Baseline Characteristics**

| **Characteristics** | **Intervention Arm** | **Control** | **Overall** |
| --- | --- | --- | --- |
| **Sample Size** (N) |  |  |  |
| **Gender** | N male (%)  N female (%)  N other (%) | N male (%)  N female (%)  N other (%) | N/A |
| **Age**  Mean (SD) |  |  | N/A |
| **Race/Ethnicity** | American Indian/Alaska Native: N (%)    Asian: N (%)    Black or African American:  N (%)    Native Hawaiian/Other Pacific Islander: N (%)    Hispanic or Latino: N (%)    White: N (%) | American Indian/Alaska Native:  N (%)    Asian: N (%)    Black or African American:  N (%)    Native Hawaiian/Other Pacific Islander: N (%)    Hispanic or Latino: N (%)    White: N (%) | N/A |
| **Setting** | *Where is the study taking place (eg. inpatient, outpatient, home based, telephone/ video conferencing, combination of multiple settings, etc...)?* | *(ex. inpatient, outpatient, home based, combination of multiple settings, etc...)* | *(ex. inpatient, outpatient, home based, combination of multiple settings, etc...)* |
| **follow-up timepoints and duration** | *At what time points were patient reported outcomes collected (eg. baseline, 3 months, 6 months, etc…)?* |  |  |
| **Type of cancer** | *What type of cancer are the participants of the study suffering from (eg. Breast cancer, “advanced malignancies,” etc…)?* |  |  |
| **Did the study take place in the perioperative period?** | *Yes/No*  *The perioperative period is defined as 30 day before and up to 90 days after a surgical procedure.*^1^ | *Yes/No* | *Yes/No* |
| **Did the study take place around the End-of-Life?** | *Yes/No*  *The End-of-Life period is defined as the last 6 months of a patient’s life or if the patient has a prognosis to live for 6 months or less.*^2,3^ *Hospice care will also be considered as the End-of-Life period.*^4^ | *Yes/No* | *Yes/No* |
| **Type of treatment** | *If yes, mention the types of surgeries/ treatment the patients underwent.*  *If no, write N/A* | *If yes, mention the types of surgeries/ treatment the patients underwent.*  *If no, write N/A* | *If yes, mention the types of surgeries/ treatment the patients underwent.*  *If no, write N/A* |

**Interventions**

Overall Directions: *Some boxes will not be applicable to certain studies, and thus can be filled in with “N/A.”*

| **Question** | **Intervention Arm** | **Control** |
| --- | --- | --- |
| **Arm description** |  |  |
| **Were APPs (includes PAs, NPs, and CNSs) involved in the study?** | *Yes/ No*  *APPs include physician assistants (PAs), nurse practitioners (NPs), certified nurse midwives (CNMs), and certified registered nurse anesthetists (CRNAs).*^5^ | *Yes/ No* |
| **Were RNs involved in the study?** | *Yes/ No* | *Yes/ No* |
| **Were social workers involved in the study?** | *Yes/ No* | *Yes/ No* |
| **What was the role of the non-physicians?** | *Write out the roles that the non-physicians were responsible for. Also include what specific type of APP were included if applicable (PAs, NPs, or CNSs)* | *Write out the roles that the non-physicians were responsible for.* |
| **What were the primary patient reported outcomes?** | *List all the patient reported primary outcomes that were collected in the study. These are the outcomes that the research study is designed to specifically measure.*^6^ *If the article does not distinguish between primary and secondary outcomes, include all patient reported outcomes as primary outcomes.* | *List all the primary patient reported outcomes that were collected in the study. These are the outcomes that the research study is designed to specifically measure.* |
| **What were the secondary patient reported outcomes?** | *List all the patient reported secondary outcomes that were collected in the study. These are the outcomes that the research study is not designed to specifically measure, but are collected by the research team and are related to the primary outcome.*^6^ | *List all the secondary patient reported outcomes that were collected in the study. These are the outcomes that the research study is not designed to specifically measure, but are collected by the research team and are related to the primary outcomes.* |
| **Did patient reported outcomes improve?** | *If yes, list which outcomes improved and designate if significant improvement.*  *If not, list the major outcomes that were measured and indicate there was no significant improvement.* | *If yes, list which outcomes improved and designate if significant improvement.*  *If not, list the major outcomes that were measured and indicate there was no significant improvement.* |
| **Involvement of technology** | Were telephone calls used to facilitate this intervention?  Were other aspects of technology used?  List the elements that were used:      *Examples include video communication, EHR, decision support technology, automated symptom monitoring/patient-reporting, other* | Were telephone calls used to facilitate this intervention?  Were other aspects of technology used?  List the elements that were used:      *Examples include video communication, EHR, decision support technology, automated symptom monitoring/patient-reporting, other* |
| **Did the non-physicians contact the patient throughout the intervention?** | *If yes, describe if it was on a regular schedule or a prompted contact and which team members were responsible for contacting the patient.*  *If no, write N/A.*  *Contact with the patient throughout the intervention means that the healthcare team member met with the patient at least 2 different times over the course of the intervention.* | *If yes, describe if it was on a regular schedule or a prompted contact and which team members were responsible for contacting the patient.*  *If no, write N/A.* |
| **Were the non-physicians responsible for attending any training/educational sessions?** | *If yes, describe the training and the frequency of the training (or if it was the just time).*  *If no, write N/A.* | *If yes, describe the training and the frequency of the training (or if it was the just time).*  *If no, write N/A.* |
| **What other healthcare team members were involved?** | *List out the different healthcare team members involved and what role they played.* | *List out the different healthcare team members involved and what role they played.* |
| **What were the team structures?** | *Include whether the healthcare team was working towards a common goal, the number of roles on the team (and each person’s responsibilities).* | *Include whether the healthcare team was working towards a common goal, the number of roles on the team (and each person’s responsibilities).* |
| **What were the team processes?** | *Explain any form of communication/ collaboration that occurred with the healthcare team. (eg. whether they had consistent meetings, ways they communicated, etc…)* | *Explain any form of communication/ collaboration that occurred with the healthcare team. (eg. whether they had consistent meetings, ways they communicated, etc…)* |
| **Were families/ surrogate decision makers/ caregivers involved in the patient’s healthcare?** | *If yes, included how they were involved.*  *If no, write N/A*  *Were family members, caregivers, and/or surrogate decision makers involved in the intervention (eg. receiving training, having follow-ups with nurses, etc… ) or involved in making any healthcare decision with the patient?* | *If yes, included how they were involved.*  *If no, write N/A* |

**Outcomes**

Overall Directions: Each study will likely have different outcomes and different ways they are reporting the outcomes, thus, individual tables will have to be built for each article (some articles might have multiple usable outcomes and will have multiple tables that we will extract).

- Outcome name: *Include the different types of patient reported outcomes (quality of life, depressive symptoms, anxiety symptoms, etc…).*
- Outcome type: Continuous, Dichotomous, Adverse event
- Reported as: Confidence intervals (mean, CI, N), Standard deviation (mean, SD, N), Standard Error (mean, SE, N)
- Outcome group: *usually not applicable*
- Scale: *enter the name of scale, i.e FACT-G*
- Range: *enter the possible range of results, i.e. 0-10*
- Unit of measurement: *usually not applicable*
- Direction*:* Lower is better or Higher is better
- Data value: Is the change calculated from baseline or endpoint (*usually change is calculated from baseline*)
- Notes: *anything else that helps us better understand the results*

- *Save as an individual outcome. Enter the timepoints of follow-up (i.e. Baseline and 9 months)*
- *Add a new outcome for different types of patient reported outcomes if applicable in the study*

**Appendix S3:** Risk of Bias of Included Studies

| **Study** | **Sequence Generation** | **Allocation Concealment** | **Blinding of Participants and Personnel** | **Blinding of Outcome Assessors** | **Incomplete Outcome Data** | **Selective Outcome Reporting** | **Other Source of Bias** |
| --- | --- | --- | --- | --- | --- | --- | --- |
| Inman et al., 2011 | Low | High | High | High | Low | Low | Low |
| Koinberg et al., 2004 | Low | Low | High | High | Low | Low | Low |
| Malmstrom et al., 2016 | Low | Low | High | High | Low | Low | Low |
| McCorkle et al., 2009 | Low | Unclear | High | High | Low | Low | Unclear |
| Koet et al., 2021 | Low | High | High | Unclear | Low | Low | High |
| Verschuur et al., 2009 | Low | High | High | High | Low | Low | Low |
| Mertz et al., 2017 | Low | High | High | High | Low | Low | Low |
| Sussman et al., 2017 | Low | Low | High | High | Low | Low | Low |
| Li et al., 2016 | Low | High | High | High | Low | Low | Low |
| Harrison et al., 2011 | Low | High | High | Low | Low | Low | Low |
| Quist et al., 2018 | Low | High | Low | Low | Low | Low | Low |
| Wallen et al., 2012 | Low | Unclear | High | Low | Low | Low | Low |
| Zhou et al., 2020 | Low | Low | Low | Low | Low | Low | Low |
| Zhu et al., 2021 | Low | Unclear | Unclear | Unclear | Low | Low | Low |
| Zheng and Jiang, 2019 | Low | Unclear | High | Unclear | Low | Low | Low |
| Young et al., 2013 | Low | Low | high | Low | Low | Low | Low |
| Watson et al., 1988 | Low | Unclear | High | Unclear | Low | Low | Low |
| Temur and Kapucu, 2019 | Low | Low | High | Unclear | Low | Low | Low |
| Sui et al., 2020 | Low | Low | High | Unclear | Low | Low | Low |
| Xu et al., 2017 | Low | Unclear | High | Unclear | Low | Low | Low |
| Ross et al., 2005 | Low | Low | High | Unclear | Low | Low | Low |
| Boesen et al., 2005 | Low | Low | High | Unclear | Low | Low | Low |
| Francke et al., 1997 | Low | Low | High | Unclear | Low | Low | Low |
| Allard 2007 | Low | Low | High | Unclear | Low | Low | Low |
| Bjerring et al., 2020 | Low | Low | Unclear | Unclear | Low | Low | Low |
| Bahrami et al., 2012 | Low | High | High | Unclear | Low | Low | Low |
| Ohlsson-Nevo et al., 2017 | Low | Low | High | Unclear | Low | Low | Low |
| Ding et al., 2022 | Low | Low | Low | Unclear | Low | Low | Low |
| Hu et al., 2022 | Low | Low | Unclear | Unclear | Low | Low | Low |
| Ji et al., 2022 | Low | Low | Unclear | Unclear | Low | Low | Low |
| Zhao et al., 2021 | Low | Low | Unclear | Unclear | Low | Low | Low |
| Turkdogan et al., 2022 | Low | Low | Low | Low | Low | Low | Low |
| Yu et al., 2022 | Low | Low | Low | Unclear | Low | Low | Low |
| Yuan et al., 2022 | Low | Low | Unclear | Unclear | Low | Low | Low |

**Appendix S4:** Intervention Purpose and Results of 27 Included Studies

| **Study** | **Intervention Purpose** | **Intervention Description** | **Results of the Studies with MCIDs** |
| --- | --- | --- | --- |
| Inman et al., 2011 | Understand the impact of follow-up educational telephone calls on the patient’s healthcare understanding after radical prostatectomy. | “Experienced urology nurses [used] an algorithm for educational reinforcement. The patient was first queried generally (“How are you doing?”) and followed up with a second query (“Have you had any problems or concerns since you were dismissed?”) if there were no initial concerns expressed. If the patient identified education needs, reinforcement was provided.” | **No results with MCID reported.** The article did state that “it is significant that the control group had additional questions about their care and was using call back opportunities to obtain further information about their condition when information had been provided in written materials and in verbal education.” |
| Koinberg et al., 2004 | Understand the impact of nurse-led follow-up on the patient’s anxiety after breast cancer surgery. | “At a visit to the physician that took place following radiotherapy and after randomisation, patients were given an appointment to meet with an experienced nurse approximately three months after surgery. Int he course of this meeting, the patient received information about how to recognise a recurrence in breast, skin, axilla and scar. The [CNS] arranged mammography at 1-year intervals and informed about the result of the mammography by telephone or letter. After 3 years, the patients were referred back to the routine mammography-screening programme. The [CNS] gave advice on aspects of self-care, such as medication and breast self-examination and provided time for talking about the patient’s psychosocial situation. The patient was requested to contact the [CNS] as soon as she had any questions or symptoms that she perceived could be related to breast cancer. The [CNS] coordinated the healthcare resources and consulted a physician or a physiotherapist when needed. The [CNS] worked in a setting where she had rapid access to specialists in surgery and/or oncology within her own hospital.” | Using the HADS, the number of months patients had anxiety and depression were assessed. There were **no clinically significant differences as determined by the MCID** in relation to anxiety and depression between the groups.   - 5.6% of the control arm and 9.7% of the intervention arm had anxiety for 6 out of 60 months with a relative risk of 1.8 and a 95% confidence interval of 0.7–4.8. - 0.8% of the control arm and 0.8% of the intervention arm had depression for 6 out of 60 months with a relative risk of 1.0 and a 95% confidence interval of 0.6-16.4. |
| Malmstrom et al., 2016 | Understand the effect of a nurse-led telephone supportive care program on the patients' quality of life after esophageal cancer surgery. | “The [nurse-led telephone supportive care program] included a meeting before discharge where the patients had the opportunity to ask questions, discuss their concerns and [receive] both oral and written information focusing on life after surgery, self care, plans for the future, and where to turn to for help if needed.” | Using the QLQ-C30, patient function and symptoms were assessed. **The only category that had a clinically significant difference as determined by the MCID was dyspnea.** All the other categories for function and symptoms did not have a clinically significant difference.   - Patients in the intervention arm had a mean of 35.9 and a standard deviation of 29.7 while patients in the control arm had a mean of 18.8 and a standard deviation of 16.9 at the 6 month follow-up for QLQ-C30 for dyspnea (p = 0.041). |
| McCorkle et al., 2009 | Understand the effects of a nursing intervention on quality of life outcomes in post-surgical women with gynecological cancers. | “Patients in the nursing intervention group received 6 months of tailored specialized care by an oncology Advanced Practice Nurse (APN). The primary objective of the intervention was to assist  patients in developing and maintaining self-management skills post-operatively and to facilitate their active participation in decisions affecting their subsequent treatment, which included chemotherapy. APN activities included symptom management and monitoring, emotional support, patient education, coordination of resources, referrals, and direct nursing care. Services included 18 patient contacts during the first 6 months after hospital discharge. The plan of care and intervention  strategies were individually tailored to each patient’s needs and personal priorities and were determined jointly by the nurse and patient. For example, while all patients received post-surgical  wound care and medication management, at the first contact one patient might need detailed instruction about nutrition while another might prioritize spiritual concerns. At subsequent contacts, patient needs and prioritizes might, for example, shift to concerns about family members, issues of sexuality, pain management, and/or side effects of chemotherapy. At baseline assessment, patients were screened for emotional distress using the Distress Thermometer (DT). Women randomized to the  intervention group who scored four or greater on the DT, indicating significant distress, received an evaluation by the psychiatric consultation–liaison nurse (PCLN). The PCLN assessed the patient’s emotional needs and screened for psychiatric disorders as recommended by the National Comprehensive Cancer Network (NCCN) guidelines. After the first PCLN contact, the APN and PCLN reviewed the plan of care in collaboration with the patient. Patients in both the intervention and attention control groups received the Symptom Management  Toolkit (SMT), a manual written at the 6th grade level with information on 16 symptoms commonly experienced post-surgically or with chemotherapy. Each section describes causes of symptoms, strategies for managing symptoms, and  when to call the oncologist.” | Using the Mishel Uncertainty in Illness Scale (MUIS), the SDS, and the SF-12, results regarding the patient’s quality of life were assessed. **The MUIS had a statistical significance**, but the MCID has not yet been created. **The SDS had a statistical significance but not a clinically significant difference based on the MCID**. The SD-12 did not have a statistically or clinically significant difference.   - For the MUIS, patients in the intervention arm had a mean of 31.1 and a standard deviation of 10.6 while patients in the control arm had a mean of 27.9 and a standard deviation of 10.5 at the 6 month follow-up. - For the SDS, patients in the intervention arm had a mean of 22.9 and a standard deviation of 6.8 while patients in the control arm had a mean of 19.9 and a standard deviation of 5.1 at the 6 month follow-up. - For the SF-12 Physical, patients in the intervention arm had a mean of 41.0 and a standard deviation of 11.4 while patients in the control arm had a mean of 44.8 and a standard deviation of 11.9 at the 6 month follow-up. |
| Koet et al., 2021 | Understand the effects of preoperative group education on physical and role functioning in patients with colorectal cancer. | “The group education [consisted] of a 1-h session. This session  [took] place after the preoperative consultation at the outpatient  clinic and at least 1 week prior to surgery. In this session,  participants and directly involved caregivers received information by using a PowerPoint presentation given by the colorectal nurse practitioner. The first part of the group education concentrated on etiology, diagnosis, therapy, hospitalization, potential side effects, out of hospital recovery, and postoperative functional implications of colorectal cancer and its surgical interventions. The second part consist[ed] of the psychoeducation part in which coping with cancer and potential practical, social, and relational problems are addressed. The last part of this meeting consist[ed] of a group discussion. Prior to this study, all collaborating professionals (doctors,nurses, dieticians, physiotherapists) received the same  educational session to ensure that the patient is approached  in a similar mindset receiving similar information at all times." | Using the QLQ-C30, patient function and symptoms were assessed. **The only categories that had a clinically significant difference as determined by the MCID were global health status and body image.** All the other categories for function and symptoms did not have a clinically significant difference.   - For QLQ-C30 for global health status, patients in the intervention arm had a mean of 72.1 (95% confidence interval: 65.7–78.5) while patients in the control arm had a mean of 63.9 (95% confidence interval: 57.7–70.0) at the 1 month follow-up (p = 0.047). - For QLQ-C30 for body image, patients in the intervention arm had a mean of 96.1 (95% confidence interval: 90.5–101.7) while patients in the control arm had a mean of 85.5 (95% confidence interval: 79.6–91.3) at the 1 month follow-up (p = 0.010). |
| Verschuur et al., 2009 | Understand the effects of nurse-led follow-up on quality of life outcomes in patients after esophageal or gastric cardia cancer surgery. | “Nurse-led follow-up was performed by home visits of a specialist nurse with more than 10 years experience in oncological care. Didactic training included a syllabus on diagnosis and treatment of esophageal and gastric cardia cancer, potential problems after esophageal resection and medical-legal issues. Standard follow-up was performed by a group of two senior surgeons at the outpatient clinic of the Erasmus MC Rotterdam and one senior surgeon at the Reinier de Graaf Hospital Delft. The participating surgeons as well as the specialist nurse filled out standardized case record forms. Case record forms include a list of items for assessment of patients during the follow-up visits, such as experienced problems and symptoms, body weight and the ability to eat and/or swallow using a dysphagia score. During follow-up, all patients were discussed during 4-weekly multidisciplinary meetings in the participating hospitals. Scheduled follow-up visits for both follow-up groups were 6 weeks, and3, 6, 9 and 12 months after randomization.” | Using the QLQ-C30, patient function and symptoms were assessed. There were **no clinically significant differences as determined by the MCID.**   - For the QLQ-C30 pain scale, patients in the intervention arm had a mean of 22 and a standard deviation of 12 while the patients in the control arm had a mean of 22 and a standard deviation of 14 at the 13 month follow-up (p = 0.24). - For the QLQ-C30 physical functioning scale, patients in the intervention arm had a mean of 82 and a standard deviation of 8 while the patients in the control arm had a mean of 78 and a standard deviation of 9 at the 13 month follow-up (p = 0.45). |
| Mertz et al., 2017 | Understanding the effects of individually tailored nurse navigation on patient-reported psychological and physical symptoms in patients with newly diagnosed breast cancer. | “The aim of the intervention was to improve patient-reported psychological and physical symptoms by symptom screening and counseling with a clinical nurse navigator. In addition to the questionnaire measurements at baseline, 6 and 12 months, patients in the intervention group provided information on patient-reported psychological and physical symptoms (distress thermometer, HADS and QLQ-C30) in screenings at 1, 9 and 18 weeks after surgery. A positive screening was defined as scores above the norm of 7 on the distress thermometer, of 8 on anxiety and depression or the various norms on health-related quality of life at any of the four assessment times and provided the basis for dialog between the patient and the nurse navigator. Navigation was performed by one experienced nurse specialized in [breast cancer] and included individual, manual-based counseling based on strategies from cognitive therapy and psychoeducation to motivate and support patients in self-managing their symptoms, and using existing rehabilitation services at the hospital or at the Copenhagen Centre for Cancer and Health, which is the local rehabilitation center. The first session was conducted face-to-face, while the following sessions were either face-to face or by telephone depending on individual need. Each session included: (1) empathetic listening and dialog, (2) collaborative empiricism (patient’s private world and navigator’s professional standards), (3) assessment of needs from patient-reported outcome measures and dia-log with the patient, (4) psychoeducation, (5) goal-setting, (6)intervention agreements and plan and (7) debriefing. The nurse navigator could refer the patient to up to six individual sessions with a project psychologist at the CopenhagenCentre for Cancer and Health run by the Danish CancerSociety counseling unit.” | Using the HADS and QLQ-C30, patient anxiety, depression, and function were assessed. **The categories that had a clinically significant difference as determined by the MCID were anxiety, depression, emotional functioning, and global health status.** Physical functioning did not have a clinically significant difference.   - For anxiety based on HADS, patients in the intervention arm had a mean of 5.2 and a standard deviation of 3.6 while the patients in the control arm had a mean of 7.8 and a standard deviation of 4.6 at the 12 month follow-up. - For depression based on HADS, patients in the intervention arm had a mean of 2.2 and a standard deviation of 2.9 while the patients in the control arm had a mean of 4.4 and a standard deviation of 4.0 at the 12 month follow-up. - For emotional functioning, patients in the intervention arm had a mean of 76.6 and a standard deviation of 18.6 while the patients in the control arm had a mean of 69.1 and a standard deviation of 24.0 at the 12 month follow-up. - For global health status, patients in the intervention arm had a mean of 77.5 and a standard deviation of 19.7 while the patients in the control arm had a mean of 67.5 and a standard deviation of 20.9 at the 12 month follow-up. |
| Sussman et al., 2017 | Understanding the effects of a specialized oncology nursing supportive care intervention on quality of life outcomes in patients with newly diagnosed breast and colorectal cancer following surgery. | “The intervention consisted of usual care plus referral to a standardized specialized oncology nurse service provided by the Interlink Community Cancer Nurses (ICCN) program in Toronto. This service was structured around four program components: (i) direct oncology nursing prac-tice; (ii) coordinated mobilization and linkage to services; (iii )community-based education and research; and (iv) community resource development. The program was provided by five registered nurses, overseen by a nurse manager. Most of these nurses were Masters prepared; all were certified in Oncology Nursing (CON(C)) with a minimum of 4 years specialty cancer experience. ICCN is modeled after the The ICCN model was intended to follow a tailored approach to care based on a comprehensive assessment of each patient’s needs,care requirements, and wishes. The nurses did not provide medical care but information and emotional support, as well as coordinating and navigating access to other needed supportive cancer care services. Emotional care by ICCN nurses included supportive care techniques like active listening, clarification, explanation and education, and guid-ance in coping/managing the effects of cancer. ICCN employed a Standardized Nursing Intervention Protocol (SNIP) for the nurses to follow. This protocol included comprehensive assessment, information and emotional support, and care planning specific to breast and colorectal cancers during the initial phases of treatment, with a minimum of two home visits. A training session was held with Interlink nurses prior to the study commencing to ensure consistency in providing supportive care tailored to this phase of care and in the timing of intervention follow-up.” | Using the patient reported outcome of continuity of care (CCCQ) and the QLQ-C30, patient function and symptoms were assessed. **The CCCQ had a statistical significance, but the MCID has not yet been created.** None of the QLQ-C30 had a clinically significant difference as determined by the MCID.   - For patient opinions on their level of information based on the CCCQ, patients in the intervention arm had a mean of 3.8 and a standard deviation of 0.8 while the patients in the control arm had a mean of 3.6 and a standard deviation of 0.9 at the 3 week follow-up. |
| Li et al., 2016 | Understanding the effects of a home-based, nurse-led health program on quality of life outcomes in postoperative patients with early-stage cervical cancer. | "1. Establishment of a family care team: a family care team was established consisting of specialist nurses, gynecological doctors, physiotherapists, psychological consultants, dietitians and physical therapists. A specialist nurse served as the leader and was responsible for nursing education, promoting the program,collecting scales, and carrying out the follow-up. Other team members provided consultation and advice based on their respective expertise.  2. Physiological rehabilitation: standard Kegel exercises, for pelvic floor muscle training (PFMT), were introduced specifically for this program. The training method was explained and taught before patients left the hospital.  3. Emotion-release management: a simple yoga exercise was introduced for this study. The training method was taught by our researchers,who received amateur training. Half an hour each time and twice a day were recommended. Everyone was offered an instructional CD that contained the yoga training video before leaving the hospital.  4. Informal social support systems: informal social support systems consisted of surrounding family and friends. We enhanced the education through the informal social support system. We explained the disease, alleviated worry and fear, and encour-aged giving more support. To encourage each other, we built a communication between new patients and those who received a successful NLHP-HB program.  5. Home follow-up monitoring: an online communication platform was implemented to solve questions and give advice. A telephone follow-up was carried out every two weeks, and a home visit every two or three months was available with the permission of the patients. Some education and questionnaires were finished when patients returned to visit doctors in the outpatient department." | Using the QLQ-C30, patient quality of life was assessed. **Overall quality of life for the patients had a clinically significant difference as determined by the MCID.**   - For overall quality of life based on the QLQ-C30, patients in the intervention arm had a mean of 101.07 and a standard deviation of 10.92 while the patients in the control arm had a mean of 90.07 and a standard deviation of 10.19. |
| Harrison et al., 2011 | Understanding the effect of a supportive care intervention on quality of life outcomes in people following surgery for colorectal cancer. | “The CONNECT telephone intervention comprises 5 calls following the patients initial discharge from hospital after surgery (days 3 and 10 and then at 1, 3, and 6 months). The format of each call is standardized with a set of questions acting as a screening tool, designed to address common problems experienced by patients throughout this period. Physical, psychosocial, information, supportive care, and rehabilitation needs are assessed and addressed during each call. Patients also have the opportunity to raise any additional concerns. The CONNECT intervention is supplementary to usual follow-up and is provided by entirely by telephone with no face-to-face contact. CONNECT is delivered by an experienced colorectal cancer nurse who has undergone training in telephone communication. If the nurse identifies a need, relevant information is provided. Emotional support is given when necessary. Where further clinical advice, or referral, is required, the nurse directs patients back to the appropriate clinical team member to make the relevant appointments and referrals” | Using the FACT-C, patient quality of life was assessed. **Overall quality of life for the patients had a clinically significant difference as determined by the MCID.**   - For overall quality of life based on the FACT-C, patients in the intervention arm had a mean of 106.0 and a standard deviation of 19.3 while the patients in the control arm had a mean of 98.6 and a standard deviation of 23.4 at the 6 month follow-up. |
| Quist et al., 2018 | Understanding the effect of an early initiated postoperative rehabilitation intervention on fatigue in patients with operable lung cancer. | “The postoperative rehabilitation was the same in both intervention groups and consisted of a supervised 12-week rehabilitation program containing 24 group-based exercise sessions, three individual counseling sessions, and three group-based lessons in health-promoting behavior. If the participants had special needs in terms of smoking cessation, nutritional counseling or patient education, this was offered too. The postoperative physical exercise consisted of an individually prepared supervised strength exercise and a group-based cardiovascular exercise twice a week (60 min/sessions) on non-consecutive days for 12 weeks, a total of 24 sessions.” | Using the EORTC-C30, patient symptoms were assessed. **There was a clinically significant difference as determined by the MCID for appetite loss, constipation, and fatigue.** There was no clinically significant difference for any of the other categories.   - For appetite loss, patients in the intervention arm had a mean of 4 and a standard deviation of 15 while the patients in the control arm had a mean of 11 and a standard deviation of 25 at the 26 week follow-up. - For constipation, patients in the intervention arm had a mean of 5 and a standard deviation of 12 while the patients in the control arm had a mean of 9 and a standard deviation of 21 at the 26 week follow-up. - For fatigue, patients in the intervention arm had a mean of 28 and a standard deviation of 19 while the patients in the control arm had a mean of 35 and a standard deviation of 21 at the 14 week follow-up. |
| Wallen et al., 2012 | Understanding the effects of a multidisciplinary consult team intervention on palliative care outcomes in surgical oncology patients with advanced malignancies. | “The PPCS is a consult team available to all patients who are actively participating in research studies throughout the NIH, CC. There are over 6,000 patient visits per year made by this team. The patients are seen as inpatients as well as in an outpatient clinic setting. The team was established in August of 2000 and at the time of the study had two full time attending physicians, three nurse practitioners, a nurse thanatologist (member of the team who specialized in the psychosocial and emotional aspects of death and dying), and one physician fellow in Hospice and Palliative medicine. The extended team included spiritual ministry, social work, recreation therapy, counseling, nutrition, acupunc-ture, acupressure, massage, reiki, rehabilitation medicine. Each consult included a full assessment of pain and other symptoms, what treatments had been implemented, and what were the most bothersome and disruptive to the patient. The consult covered not only physical symptoms, but emotional and spiritual distress. The patient was usually offered varied modalities of treatment, pharmacologic as well as complementary therapies. The philosophy of the team was to provide comfort care for symptom burden earlier in the disease process to improve quality of life.” | Using the SDS, Gracey Pain Scale, and Center for Epidemiologic Studies Depression Scale, patient pain and symptoms were assessed. **There was a statistically significant difference in pain unpleasantness, but no MCID has been created yet.** There was no clinically significant difference for any of the other categories.   - For pain unpleasantness, patients in the intervention arm had a mean of 6.73 and a standard deviation of 3.71 while the patients in the control arm had a mean of 6.71 and a standard deviation of 3.86 after the surgery and intervention. |
| Zhou et al., 2020 | Understanding the effects of a WeChat-based multimodal nursing program on early rehabilitation in postoperative women with breast cancer. | "Patients in the intervention group received WeChat-based multimodal nursing program plus routine nursing care. The WeChat-based program group included 1 doctor and 1 nurse from the surgical breast cancer department, 3 researchers, and 1 postgraduate trainee majoring in breast cancer care. All members participated in the program design; 2 members were responsible for information preparation and delivery; and 3 members (WW, PG, and CZ) handled problems arising on the WeChat platform. The duration of the intervention spanned the continuous period between hospital admission and 6 months post-surgery. To avoid between-group contamination, the researchers (PG and WW) placed the patients in separate areas of the surgical breast cancer department, with no contact allowed between the groups. Staffing was also specific to each group with no overlapping personnel allowed. Physical rehab, psych rehab, social rehab and implementation [also occurred]." | Using the FACT-B, patient well-being was assessed. **There was a clinically significant difference as determined by the MCID for total well-being.**   - For total well-being, patients in the intervention arm had a mean of 106.00 and a standard deviation of 11.73 while the patients in the control arm had a mean of 98.42 and a standard deviation of 16.59 at the 1 month follow-up. |
| Zhu et al., 2021 | Understanding the effect of evidence-based nursing on anxiety and depression in patients with gastric cancer. | “The evidence-based nursing group was composed of the responsible nurse, the nurse in charge and the head nurse. The head nurse served as the team leader and was responsible for supervising and managing the work of the team, while the team members were responsible for consulting the relevant literature, determining the difficulties and priorities of nursing cooperation, and formulating nursing measures. Cooperation of itinerant nurses: Based on the tumor size, location and surrounding organ invasion of patients, the relevant instruments, including sterile distilled water, medical bio glue and surgical film, were prepared, and the shadow less lamp lighting was adjusted in time to obtain a clear surgical field, keep the suction tube unobstructed, timely suck out the bleeding and exudate, and reduce the exfoliated cancer cells, To avoid contamination of abdominal cavity, and to observe the operation process closely, timely replace the contaminated dressings and instruments Equipment nurse cooperation: strictly distinguish the “tumor free area” and “tumor area”, keep the gauze dry in the cancer area, use gauze pad to protect the surrounding tissues when the tumor is removed, and timely handle the gloves and gauze contaminated by the tumor. In addition, the instruments used before and after tumor resection should be placed separately, and the removed lymph nodes should not be directly contacted with hands. The lymph nodes should be arranged on the moist gauze. After the tumor was removed and the digestive tract was reconstructed, the surgical instruments were replaced. Before closing the abdomen, the abdominal cavity was rinsed with 1000-3000 ml distilled water for 2-4 times, and the residual liquid was sucked out with aspirator to prevent infection.Prevention of complications: during the operation, oxygen saturation and vital signs of the patients were closely observed, the distribution of large vessels in the upper abdomen was understood, and the methods of vascular anastomosis or repair were mastered. First aid items should be prepared before operation, such as vascular suture, artery clip and rubber sling. In case of vascular injury during operation, timely cooperate with doctors for vascular anastomosis or repair.” | Using the SAS and SDS, patient anxiety and depression were assessed. **There was a clinically significant difference as determined by the MCID for both anxiety and depression.**   - These results were significant both 1 and 3 days after the operation. The exact results were not provided as numeric values in the study. |
| Zheng and Jiang, 2019 | Understanding the effects of a rapid recovery nursing model on perioperative indicators and unhealthy emotion after surgery in patients with lung cancer. | “The study group adopted the rapid recovery nursing model in which patients received health education since clinical treatment will be compromised when they may be afraid, uptight and nervous due to lack of understanding [of] their state of illness. Therefore, paramedics [explained] the etiopathogenesis, treatment principle and possible complications of lung cancer, and inform[ed] patients the importance of reasonable diets, quitting smoking and alcohol, as well as proper exercises. Next, paramedics communicate[d] with patients actively to establish an amicable relation, and encourage[d] them psychologically with successful cases, in order to improve their confidence and compliance in treatment. Patients’ question[s] [were] answered patiently to relieve them from any psychological stress. Analgesia [was] performed during surgery with propofol at controlled injection rate and dose; liquids [was] stored in [an] incubator, and temperature in the operating room [was] properly adjusted to copy with [the] body. Dietary intervention include[d] a little liquid on the day 1, semiliquid on days 2 and 3, and reduced fluid infusion after surgery. 6 h after surgery, patients [were] assisted for exercises by lifting their upper body properly and turning them over as their vital signs become stable, and 10 h after surgery, they practice[d] coughing and breathing. On day 1 after surgery, patients [were] required for proper exercises, including four limbs at horizontal positions, breathing and coughing at an acceptable and tolerable intensity, to improve metabolic functions, immunity and resistance, and promote recovery greatly. For instructions upon discharge, patients [were] informed of the notes and possible problems, and requirement for regular follow-up.” | Using the SDS and SF-36, patient function and symptoms were assessed. **There were clinically significant differences as determined by the MCID for the SDS score and all SF-36 categories.**   - For emotional function, patients in the intervention arm had a mean of 87.3 and a standard deviation of 11.12 while the patients in the control arm had a mean of 66.5 and a standard deviation of 9.3 after the intervention. - For social function, patients in the intervention arm had a mean of 86.5 and a standard deviation of 7.8 while the patients in the control arm had a mean of 71.6 and a standard deviation of 6.9 after the intervention. - For role function, patients in the intervention arm had a mean of 84.8 and a standard deviation of 8.2 while the patients in the control arm had a mean of 73.1 and a standard deviation of 7.8 after the intervention. - For cognitive function, patients in the intervention arm had a mean of 83.8 and a standard deviation of 7.3 while the patients in the control arm had a mean of 69.6 and a standard deviation of 5.8 after the intervention. - For SDS, patients in the intervention arm had a mean of 25.3 and a standard deviation of 2.1 while the patients in the control arm had a mean of 37.6 and a standard deviation of 2.7 after the intervention. |
| Young et al., 2013 | Understanding the effect of a centralized nurse-led telephone-based care coordination intervention on patient-reported outcomes in patients after surgical resection for colorectal cancer. | “[The] CONNECT [intervention] aims to improve patients’ ability to navigate the health system for clinical and supportive care, as well as to provide information and emotional support directly, so as to improve patients’ cancer-related QOL. CONNECT is supplementary to usual follow-up care and involves no face-to-face contact. It consist[ed] of five scheduled, structured telephone calls on days 3 and 10 and thenat 1, 3, and 6 months after hospital discharge, based on the findings of a clinical audit of postoperative needs of patients with colorectal cancer. Each call include[d] 22 standardized screening questions about common physical, psychosocial, information, supportive care, and rehabilitation/follow-up needs. In addition, at 1 month, patients with stage C colon cancer were asked if they had discussed the option of adjuvant chemotherapy with their physicians; if not, they were prompted to ask about this during their next consultation. Identified needs were addressed by the intervention nurse using detailed, standardized clinical protocols according to the nature and severity of the need and level of clinical risk posed. For low-risk needs, the nurse provided relevant information and advice so that the patients could seek appropriate assistance from their local care providers. For a serious or potentially high-risk problem(eg, suicidal ideation), the intervention nurse contacted a member of thepatient’s local health care team directly. The intervention nurse did not make independent referrals to other health professionals but linked patients back to their local health team for appropriate referrals to be made. After random assignment, general practitioners for patients allocated to the intervention group were informed about the CONNECT intervention so that they were aware of these processes. The intervention was delivered by experienced nurses who received training and ongoing debriefing by senior researchers with backgrounds in nursing, psychology, and medicine. Ten percent of telephone calls (randomly selected) were audiotaped to monitor and maintain intervention fidelity. In-tervention nurses were employed specifically for this study and were located centrally within the research office. They had no contact with patients or clinicians as part of standard clinical care at any of the participating hospitals.Intervention nurses received the contact details only of patients allocated to receive the CONNECT intervention and were unaware of patients in the control group. Together with the nondisclosure of group allocation until after hospital discharge, these measures ensured that the risk of contamination between groups was negligible.” | Using the FACT-C, patient quality of life was assessed. **There were no clinically significant differences as determined by the MCID for the patient reported outcomes**, however, the study states “Quantitative questionnaire responses (n=350) about the CONNECT nurse and intervention were consistently positive.” |
| Watson et al., 1988 | Understanding the effects of a specialist nurse counseling intervention on depression in patients with breast cancer. | “An individual counseling service was offered to patients immediately [after] the diagnosis was known, with the nurse being present when the doctor informed the patients of the diagnosis. The counselor would subsequently make a home visit prior to admission and would then see the patients after admission and before surgery. They would be seen on at least one occasion postoperatively prior to discharge from hospital. This was followed by a home visit some 2 or 3 weeks later. Up to this point all new patients were seen, then following the postoperative home visit, the service continued ‘on demand’, with the nurse-counselor seeing patients at follow-up clinics to check on progress. If a recurrence of the cancer was diagnosed at a later date, counseling would recommence with patients being seen during the course of the clinic at which recurrence was confirmed. Where needed counseling was available throughout the terminal stage of the illness. Patients were seen by the same counselor (SD), thereby enabling a continuous relationship to develop. During counseling the diagnosis was discussed openly and this was considered an important part of gaining patients’ trust. The counseling process followed a general framework for all patients and covered three areas: (1) emotional support and facilitation of adjustment (2) information about physical state, and (3) practical advice on breast prostheses.” | Using the Profile of Mood States and patient reported questions, patient mood and symptoms were assessed. **There was a statistically significant difference in health related problems, but no MCID has been created yet.**   - For health related problems, patients in the intervention arm had a mean of 4.0 and a standard deviation of 1 while the patients in the control arm had a mean of 5.1 and a standard deviation of 2 after the intervention. |
| Temur and Kapucu, 2019 | Understanding the effect of the Self-Management of Lymphedema Program on quality of life outcomes in patients with breast cancer-related lymphedema. | “The researcher informed the patients for the Self-Management of Lymphedema Program (SMLP), and its objective and patients were followed for six months. Within the first 24 h after the surgery, patients were asked to do hand squeezing exercises with a medium-level stress ball four times a day, squeezing 15 times each time. The minimum amount of exercises required for the patient and the time to make the patient start exercising were determined by the doctor and nurse together taking into consideration the physical condition of the patient, and the patient's general well-being during the post-operative period. In addition, patients were asked to do active and passive arm exercises three to six times a day at first, and then gradually increase the number to 10, from passive to active within 30–60 min. Before starting to exercise, patients were advised to do warm up movements to prevent any muscle damage and to divide the exercises into 10–15 min sets in case of any pain.” | Using the QLQ-C30, patient function was assessed. **There were clinically significant differences as determined by the MCID for physical, role, emotional, cognitive, and social functioning.**   - For physical functioning, patients in the intervention arm had a median of 100.0 while the patients in the control arm had a median of 40.0 at the 6 month follow-up. - For role functioning, patients in the intervention arm had a median of 100.0 while the patients in the control arm had a median of 16.7 at the 6 month follow-up. - For emotional functioning, patients in the intervention arm had a median of 91.7 while the patients in the control arm had a median of 16.7 at the 6 month follow-up. - For cognitive functioning, patients in the intervention arm had a median of 100.0 while the patients in the control arm had a median of 83.3 at the 6 month follow-up. - For social functioning, patients in the intervention arm had a median of 100.0 while the patients in the control arm had a median of 16.7 at the 6 month follow-up. |
| Sui et al., 2020 | Understanding the effect of a WeChat app-based education and rehabilitation program on anxiety, depression, and quality of life in patients with non-small cell lung cancer who underwent surgical resection. | “This study consisted of two stages: interventional stage (12 months) which was defined as the duration from the initiation of WERP to the end of WERP, and non-interventional follow-up stage (another 48 months) which was defined as the duration from the end of WERP to the patients’ death or to the completion of another 48-month follow-up (total 60 months). During the interventional stage, the WERP was administered to the patients in the WERP group for 12 months. During the non-interventional follow-up stage, patients in the WERP group were further followed up by WeChat, telephone or clinic visit (if patients needed a clinic appointment, they could inform the trained nurses in the WeChat Group, then the trained nurses made a clinic appointment for them). Besides, all patients were given conventional treatment based on the basic assessment of disease by treating physicians according to the clinical practice guidelines of NSCLC, which included postoperative infection prevention, prevention of tumor recurrence and complications, rehabilitation of emotional disorders and so on. All trained nurses supported the patients via the WeChat app apart from their daily work. At 4–8 weeks post operation, the WERP was conducted by the trained nurses who received a one-month special training related to the research contents. And the implementation of WERP was based on a mobile application (app): WeChat (Tencent Corporation, Guangzhou,Guangdong Province, China). The WERP was performed for a total of 12 months, which comprised of the following four components (Details are shown inTable 1): (i) disease-related health education, (ii) re-habilitation exercise guidance, (iii) daily activity supervision, (iv)psychological support.(i)Disease-related health education:at the first week after enrollment,patients were invited to attend a session, during which, the trained nurses introduced the WERP in detail; then a disease-related health education manual was distributed to each patient; subsequently, aWeChat Group in the WeChat app was built up by the trained nurses, and patients who were recruited in the study at the same month were invited into the same WeChat Group. After the establishment of the WeChat Group, the trained nurses delivered the disease-related health educational courses into the WeChat Group.And according to the education manual, the trained nurses explained disease-related knowledge in detail in each course. All courses were delivered in the form of short videos and updated weekly for a total of 12 weeks. After each delivery of video courses in the WeChat Group, patients were required to watch and learn the contents of videos carefully and replied“received”when finished the course. And if there was any question about the video courses, patients could consult the trained nurses in the WeChatGroup or in one-to-one chatting model if necessary, then the trained nurses would answer them in time. In addition, if patients needed a clinic appointment, they could inform the trained nurses in the WeChat Group, then the trained nurses made a clinic ap-pointment for them. (ii)Rehabilitation exercise guidance:when the weekly delivery of edu-cational courses was finished (at 12th week), the rehabilitation exercise guidance was initiated (from 13th week), which was also weekly delivered (for 40 weeks) in the form of short video courses in the WeChat Group. In each video course, the trained nurses demonstrated the exercise posture in detail and emphasized the key points of the posture as well as the points for attention. Also, if patients had any question related to the video courses, counseling in the WeChat Group or one-to-one chatting model was en-couraged.(iii)Daily activity supervision:an individualized daily walking plan was tailored for each patient, which was based on the patient's physical status, preferences as well as the advice from the treating physi-cians. And the completion of daily walking plan was measured by walked steps, which was daily counted by WeChat Movement (a step-counting database in the WeChat) and supervised by trained nurses. According to the accomplishment of daily goals over the past week, through WeChat, the trained nurses would motivate and promote (once a week) patients to fulfill the daily walking plan, and the appropriate adjustment of the walking plan was made if necessary.(iv)Psychological support:individualized nurse-led counseling via the video call on the WeChat was conducted every two weeks by the trained nurses. Counseling was based on a holistic assessment of each patient's life situation, mental status as well as potential challenges. Each patient was asked about potential problems indi"erent life domains such as physical, functional, psychological,social, and spiritual. Based on identified needs, patients were challenged with questions about the characteristics of the problems, factors that may influence the problems, and their problem management strategy. Also, strategies and solutions on identified problems were provided for them. If patients presented with ne-gative emotions, the trained nurses would communicate with pa-tients individually and help them slip negative emotions as much as possible and persuade patients to cooperate with treatments in a positive and optimistic attitude. While as for patients presented with any complex medical problems, they were advised to contact their treating oncologist in time.” | Using the HADS and QLQ-C30, patient anxiety, depression, and quality of life was assessed. **There were clinically significant differences as determined by the MCID for anxiety, depression, and global health status.**   - 19% of patients in the intervention arm had anxiety while 41% of patients in the control arm had anxiety after the intervention. - 20% of patients in the intervention arm had depression while 36% of patients in the control arm had depression after the intervention. - For global health status, patients in the intervention arm had a mean of 13.36 and a standard deviation of 15.41 while the patients in the control arm had a mean of 8.22 and a standard deviation of 16.70 after the intervention. |
| Xu et al., 2017 | Understanding the effect of a self-efficacy intervention on quality of life in patients with intestinal stoma. | “The two groups were exposed to routine nursing, where-as the intervention group was also given a 3-month self-efficacy intervention (including direct experience, alter-native experience, verbal persuasion, social and psychological support, and adjustment of the intervention measures according to the patients’ feedback), which took place once a week post-operatively in the first month (i.e., four times in total). In the second month post-operatively, the intervention group received either a call or visit twice a week, two times in total; and finally, in the third month post-operatively, the interven-tion group had one visit or call.” | Using the Stoma Self-Efficacy Scale, patient quality of life was assessed. **There were statistically significant differences in somatic function, emotional function, cognitive function, dysgraphia, and diarrhea, but no MCID has been created yet.**   - For somatic function, patients in the intervention arm had a mean of 73.33 and a standard deviation of 19.10 while the patients in the control arm had a mean of 52.62 and a standard deviation of 28.88 after the intervention. - For emotional function, patients in the intervention arm had a mean of 67.50 and a standard deviation of 21.44 while the patients in the control arm had a mean of 47.32 and a standard deviation of 26.84 after the intervention. - For cognitive function, patients in the intervention arm had a mean of 61.67 and a standard deviation of 23.63 while the patients in the control arm had a mean of 36.31 and a standard deviation of 29.41 after the intervention. - For dysgraphia, patients in the intervention arm had a mean of 25.00 and a standard deviation of 23.88 while the patients in the control arm had a mean of 54.76 and a standard deviation of 34.20 after the intervention. - For diarrhea, patients in the intervention arm had a mean of 18.33 and a standard deviation of 20.16 while the patients in the control arm had a mean of 53.57 and a standard deviation of 30.55 after the intervention. |
| Ross et al., 2005 | Understanding the effect of home visits on the well-being of patients with colorectal cancer. | “The patients in the intervention group were visited in their home five times during the first 2–3 months and visits were repeated approximately 4, 7, 11, 16, and 24 months after discharge, totalling 10 visits in all. Of the 125 patients in the intervention group, 77 patients (62%) received all 10 home visits, 34 patients (27%) had six to nine home visits, 12 patients (10%) had one to five home visits, and the remaining two patients (2%) had no home visits at all. Reasons for not receiving all 10 home visits were death, withdrawal or the patient moving to a nursing home. The project nurse visited 112 patients and a medical doctor visited the remaining 13 patients.The duration of the visits varied, but was generally close to 1 h. The visits were aimed at providing emotional and informational support and encouraging the patients to make use of their own social network to cope with the disease. Possible symptoms indicative of a relapse were discussed at each visit, but apart from that, the patients chose the topics of discussion and the setting of the visits.The visitor was not allowed to interfere with medication or other treatments, domestic help oraids, but could inform the patient where to apply for help and advise the patient to contact the physician, if necessary. Patients in the intervention group were offered telephone calls between visits, and the patients were given a telephone number at which the visitor could be reached at certain hours, if needed.” | Using the QLQ-C30 and HADS, patient anxiety, depression, function, and symptoms were assessed. There were **no clinically significant differences as determined by the MCID.**   - At the 24 month follow-up, 84% of the intervention arm and 85% of the control arm had no anxiety. - At the 24 month follow-up, 93% of the intervention arm and 94% of the control arm had no depression. |
| Boesen et al., 2005 | Understanding the effect of a psychoeducational intervention on Profile of Mood States in patients with cutaneous malignant melanoma. | “The psychoeducational intervention was offered between 3weeks and 4 months after surgery to groups of eight to 10 patients. This intervention was organized into six sessions lasting approximately 2.5 hours each and conducted over a 6-week period. Two physicians provided a health education component consisting of information about malig-nant melanoma and proper follow-up routines. Two nurses provided patients with information on cancer-preventive behavior, particularly regarding the hazards of exposure to the sun. This health education component differed from the original intervention manual in that specialized health staff and not the group therapist provided the information. The group therapist (psychologist) provided a method for stress management and a coping method. The stress management component was divided into two sections: stress awareness, during which the participants were provided with stress monitor questionnaires to increase their awareness about stress, and actual management of stress, during which patients were taught simple relaxation exercises (relaxation followed by guided imagery) and encouraged to use this technique daily by using a complementary compact disk with relaxation and imagery exercises. In the coping method component,the participants were introduced to the concepts of active and avoidance coping and effective problem solving, and asked to apply these methods in specific situations. According to the original intervention manual, the patients should discuss the different coping methods based on illustrations of coping scenarios. However, we changed this approach to discussions of the patients’ own experiences and problems on the basis of questions about various topics (eg, “How did you react when you discovered the mole?”) because the patients in the first intervention group raised objections to the scenarios in their evaluation of the intervention. Psychological support was available from two perspectives: the supportive climate provided by discus-sions among patients, and the presence of a group therapist through-out all sessions.” | Using the Profile of Mood States, patient moods were assessed. **There were clinically significant differences as determined by the MCID for total mood disturbance. There were statistically significant differences in behavior coping methods and cognitive coping methods, but no MCID has been created yet.**   - For total mood disturbance, patients in the intervention arm had a mean change of -8.43 and a standard deviation of 24.3 while the patients in the control arm had a mean change of -2.64 and a standard deviation of 22.1 after the intervention (p = 0.04). - For behavioral coping methods, patients in the intervention arm had a mean change of 1.81 and a standard deviation of 6.5 while the patients in the control arm had a mean change of -1.33 and a standard deviation of 6.4 after the intervention (p = 0.0007). - For cognitive coping methods, patients in the intervention arm had a mean change of -0.01 and a standard deviation of 6.9 while the patients in the control arm had a mean change of -3.40 and a standard deviation of 8.6 after the intervention (p = 0.0002). |
| Francke et al., 1997 | Understanding the effect of a nursing pain program on patient outcomes in patients with colon or breast cancer. | “The CE programme on pain assessment and management consisted of eight weekly 3-hour sessions. A follow-up meeting was held 4 months after the end of the programme. Program items dealt with were: (i)characteristics of pain (e.g. its uniqueness for every patient and its subjectivity) and assess-ment of pain (e.g. pain history taking and the use of rating scales); (ii) psychosocial interventions (e.g. provision of psychosocial support and information); (iii) physical and relaxation interventions (e.g. massage, and use of relaxation and distrac-tion techniques); (iv) pharmacological pain management (e.g. rationale of scheduled analgesics, and real risks of side-effects). Approximately 3 contact hours were devoted to program item (i), 7.5 contact hours to item (ii), 6 contact hours to item (iii) and 4.5 contact hours to item (iv). Remaining contact hours had a more general character (e.g. group evaluations). Elaboration of the above themes was related to the surgical-oncological background of partici-pants. This resulted in more emphasis on acute rather than chronic pain assessment and management.Various didactic strategies were used: introduc-tions by the teachers, discussions in the group as a whole and in small groups, practical exercises and provision of relevant literature.In principle, all the qualified nurses of a wardteam (including head nurses and team leaders) participated together in the program. Only in exceptional cases (e.g. protracted illness or preg-nancy) was an exception made. Coordinatinghead nurses and division managers who were not directly involved in patient care did not partici-pate. The programme took place in a room in the participating hospital and was presented by two instructors: one with a professional background in nursing and education and the other with a background in adult education.” | Using the Numerical Rating Scale for pain intensity, patient pain was assessed. **There were statistically significant differences in pain intensity, but no MCID has been created yet.**   - For pain intensity in patients with colon cancer, patients in the intervention arm had a mean of 2.9 and a standard deviation of 2.7 while the patients in the control arm had a mean of 4.7 and a standard deviation of 2.5 after the intervention on day 2. - For pain intensity in patients with breast cancer, patients in the intervention arm had a mean of 1.8 and a standard deviation of 2.1 while the patients in the control arm had a mean of 3.1 and a standard deviation of 3.0 after the intervention on day 4. |
| Allard 2007 | Understanding the effect of a psychoeducational telephone intervention on functional status and emotional distress in patients undergoing surgery for breast cancer. | “The AFSMI consisted of two phone sessions implemented on the same day of the week for two weeks. The frequency was chosen so that the patient’s recovery could be monitored on a regular basis and to facilitate the reliability of data collection and delivery of the intervention. The principal investigator was the only intervener to deliver the AFSMI. She made one phone call per week for a total of two telephone intervention sessions for each woman over a period of two weeks. The interval seemed reasonable, taking into account that the objective was to determine the efficacy of this short-term, low-cost telephone intervention. Appointments to deliver the interventions during the two weeks were made by the research assistant for patients in the experimental group after randomization. Permission to review the participants’ charts to gather information about medical status also was obtained in the consent form. The investigator performed chart review at each participating site after data collection was completed. Using the interview guide and a follow-up sheet, the investigator assessed each woman’s symptoms by asking her to identify and describe each symptom in concrete, objective terms. The actions taken by each woman to manage each symptom and the effectiveness of her actions in relieving symptoms were rated using a five-point Likert scale ranging from 1 (not effective) to 5 (very effective). Actions that women felt were effective in managing their symptoms were encouraged by the intervener. If their actions were ineffective, women were encouraged to find other potentially helpful actions. The investigator suggested new or additional self-care strategies when requested. During telephone contact, the intervener acknowledged any feelings or emotions women expressed. The length of the telephone contact was not limited to a predefined number of minutes but rather was individualized based on the number of symptoms experienced or other concerns that the women were willing to discuss.” | Using the Profile of Mood States and home management scores, patient mood was assessed. **There were statistically significant differences in home management, but no MCID has been created yet.** For the Profile of Mood States, there were no clinically significant differences as determined by the MCID.   - For home management, patients in the intervention arm had a mean of 12.12 and a standard deviation of 15.00 while the patients in the control arm had a mean of 17.10 and a standard deviation of 17.51 after the intervention. |
| Bjerring et al., 2020 | Understanding the effect of home visits by a nurse on quality of life outcomes in patients treated with self-expandable metallic stents due to incurable esophageal cancer. | “In each individual participating patient it was accepted that family members assisted in the completion of the respective questionnaires. All the patients were offered an “open admission” to the department with a Hot-Line phone number. There [was] no pre-scheduled follow-up (outside the protocol) by physicians or nurses in any of the patients but study participation did not exclude or substitute for any other services offered [to] similar patients. In general terms the home visits consisted of advice and assessment of the patient’s needs, and if it was considered relevant to make any change to the patient’s treatment, a physician could be consulted.The patients in the intervention arm had prescheduled home visits by one dedicated ward nurse after1 and 6 weeks as well as a telephone call after 11 weeks. For each patient always the same nurse completed the home visits. Each intervention (home visits) included a basic checklist of potential topics to be monitored. However, each individual visit and telephone call should focus on the topics and needs which the patient and relatives found most relevant and urgent. After each visit or telephone call the nurse completed a structured report, but otherwise no specific protocol was used. All home visits by the nurse were accepted by the patients and no patients withdrew consent during the study. Patients were assigned to palliative oncological treatment based on decisions taken at the MDT (multidisciplinary tumor team), whereupon the patients study group allocation was kept unknown.” | Using the QLQ-C30, patient quality of life was assessed. **There were clinically significant differences as determined by the MCID for the global health related quality of life (p= 0.03), dysphagia (p = 0.03), and eating disabilities (p = 0.04).**   - The exact results were not provided as numeric values in the study. |
| Bahrami et al., 2012 | Understanding the effect of a care program on pain intensity in patients with cancer who underwent surgery. | “Caring program in the experimental group consisted of 2  parts. The first part was before the surgery, including 30  min patient’s education and training that was done for the  whole group in the patients’ room. Groups consisted of at  at least 2 patients. In some cases due to the limited number  of patients, the training sessions were done individually in  their room. At the first 5 min, the aim was to introduce the intervention and empathy with the patient. This was followed with 15 min giving comprehensive information about the pain  to patients. In this part, necessary information about the meaning of the pain, different types of pain (acute, chronic, and pain related to cancer), undesirable effects of pain after surgery on physical, mental, and economic dimensions, pharmacologic and nonpharmacologic methods of pain relieving, and the method of pain measurement by different scales was given to the patient. The final part was 10 min teaching patients about breathing and relaxation methods to follow when they experience pain. At the end of the training intervention, the content of the program was given to patients in the form of a pamphlet asking them to read it. The second part of the caring program was about 5-min interaction with patients after the surgery. The content of the interaction with the patient was speaking about what was interesting for the patient, such as the experience of the surgery, learning relaxation methods, and how pain will be managed after surgery. On the first day after surgery following every interaction with the patient, pain intensity was measured by the researcher according to a numerical scale of measurement every 2 h. In addition, the patients’ complaints of pain were reported to the supervisor of the ward and analgesic was given based on what was prescribed. The amount of analgesic in every session was recorded. To prevent any interference with the patients’ sleep, pain recording was done only in the day from 8 AM to 8 PM. Totally, in the first 24 h after surgery, the patients’ pain was measured 6 times.” | Using the American Pain Society- patient questionnaire, patient pain was assessed. **There were statistically significant differences in pain intensity, but no MCID has been created yet.**   - For pain intensity, patients in the intervention arm had a mean of 1.70 and a standard deviation of 1.30 while the patients in the control arm had a mean of 2.80 and a standard deviation of 1.30 at the 24 hour follow-up after surgery (p < 0.05). |
| Ohlsson-Nevo et al., 2017 | Understanding the effect of a psycho-educational intervention on mood in patients with colorectal cancer and anal cancer. | “The setting for the PEP was the outpatient ward of a hospital surgical clinic. The sessions took place on Thursdays between the hours of 17.00 and 19.00 over seven weeks. A 60-minute lecture was followed by a one-hour discussion and reflection on the topic of the day in a group with peers. Patients were able to bring their caregivers to participate in the lectures, although patients and caregivers were separated during the subsequent group discussions. This div-ision into two groups enabled patients to speak freely about their experiences and worries without being overheard by their caregivers. The topics of the informational lectures were as follows: Colorectal cancer, Music and relaxation, The operating theater, The importance of physical activities, The meaning of food, Crisis and crisis intervention, and Patients’ organizations. The content of the PEP was influenced by ‘The family’s cancer journey’ by Kristjanson and Ashcroft, which suggested that ‘Families want information about the diagnosis, prognosis, treatment options, and expected course of recovery to lessen their fears and to increase their sense of predict-ability’. Other informational needs among cancer patients guiding the content of the PEP consisted of crisis and crisis interventions, nutrition, pain management, and the impact of cancer on the family. The programme was not part of the ordinary activities of the surgical clinic. The lectures were given by professionals who were connected to the surgical clinic. A registered nurse, who was a stoma therapist with special knowledge about colorectal as well as anal cancer, was not part of their search team but led the discussion with the patients.” | Using the MACL, patient mood was assessed. **There were statistically significant differences in overall mood and pleasantness, but no MCID has been created yet.**   - For overall mood, patients in the intervention arm had a mean of 3.15 and a standard deviation of 0.51 while the patients in the control arm had a mean of 2.95 and a standard deviation of 0.48 at the 1 month follow-up (p = 0.04). - For pleasantness, patients in the intervention arm had a mean of 3.25 and a standard deviation of 0.55 while the patients in the control arm had a mean of 2.98 and a standard deviation of 0.54 at the 1 month follow-up (p = 0.01). |
| Ding et al., 2022 | Understand the effect of nurse-led telephone follow-up care on the patients' quality of life after laryngeal cancer surgery. | “Telephone follow-ups were conducted by four specialist nurses, two nursing management graduate students, and two otolaryngology head and neck surgery graduate students assigned to the intervention and control groups. The specialist nurses were registered nurses specializing in otolaryngology head and neck surgery with (1) at least two years of experience, (2) clinical experience with laryngeal carcinoma, (3) an undergraduate college degree or above, and (4) approval to participate in the study and complete the follow-up tasks in the allotted time. Nurses in the intervention group were trained in motivational communication according to the revised NOC. All of the nurses received three training sessions and the duration of each session was 1 hour. The follow-up sequence was connect, introduce, communicate, ask and respond, and exit. (1) Connect consists of politely con-firming if the person answering the phone is the patient or a family member. (2) The nurse introduces herself and her role and states the purpose of the follow-up. (3) In communicate, the nurse obtains the patient’s consent and cooperation. (4) In ask and respond, the patient is asked the questions from the revised NOC, and the nurse provides health guidance for the patient. (5) Exit consists of the nurse thanking the patient for their cooperation and making an appointment for the next follow-up call. The traditional telephone fol-low-up focused on postoperative recovery, answered the questions raised by patients, and provided health guid-ance without unified and standardized follow-up content. Patients were followed-up at one, two, four, six, and eight weeks after discharge; thereafter, a follow-up call was made every eight weeks, which was consistent with the National Comprehensive Cancer Network and the requirements of follow-up postoperative care. A total of seven calls were made to patients in the intervention and control groups.” | Using the Functional Assessment of Cancer Therapy (FACT) –Head and Neck, patient quality of life was assessed. **There were statistically significant differences in overall patient quality of life.**   - For emotional state, patients in the intervention arm had a mean of 16.98 and a standard deviation of 1.60 while the patients in the control arm had a mean of 15.82 and a standard deviation of 1.96 (p = 0.001). |
| Hu et al., 2022 | Understand the effect of nurse-led follow-up care on the patients' quality of life after transcatheter arterial chemoembolization for primary hepatic carcinoma. | “Patients in the observation group were treated with comprehensive nursing intervention. Specific treatment included the following: (1) Before the operation, patients were informed of relevant information to allow them to fully and correctly understand the etiology, clinical manifes-tations, treatment methods, and prognosis of cancer.Any discomfort and complications that may occur after operation were explained in detail. According To the individual situation of patients, appropriate psychological care and health guidance were for-mulated, and a relationship of mutual trust with patients was established for patient encouragement and compliance as well as the elimination of negative emotions such as anxiety, fear, depression, and sadness that often appear in the treatment process. Preoperative guidance and preparation of surgical instruments and drugs were conducted, followed by a detailed physical examination and allergy tests. Patients were reminded to fast 4 hours before the operation. (2) Intraoperative: patients cooperated with physicians to complete the operation, with the indexes of patients normally checked and the internal conditions of patients monitored. Patients were informed of the chemotherapy process promptly. Physicians could remind patients to be mentally prepared before injection chemotherapy, which may cause obvious discomfort. They could also tell patients that the operation was successful and praise the patient’s compliance when it was approaching the end of the operation. Various conditions should be handled reasonably appropriately: pain care, attention to control, respiratory control, and position adjustment to relieve the patient’s pain. Patients in severe pain may be given analgesics to relieve severe pain with the consent of the physician’s diagnosis. Nursing for complications and adverse reactions was performed with prevention in advance by ventilating and maintaining a warm temperature in the ward, frequently changing sheets and clothes, closely monitoring the condition, and timely handling the side effects and complications of chemotherapy to different degrees. Diet nursing was followed according to the individual physiological and dietary charac-teristics of patients, with reasonable and healthy daily diet plans formulated, and the diet plans focused on light foods with high calorie, high cellulose, and high protein, boosting the patients’ body resistance and immunity. Daily life nursing intervention was conducted through formulating activity plans according to the actual situation of patients after operation, such as encouraging patients to get out of bed for postoperative recovery, paying attention to sleep quality, guiding patients to restmore, drinking water properly, sleeping well, carefully following the doctor’s instructions on medication, and regularly checking liver function and blood circulation.” | Using the 12-Item Short Form Health Survey (SF-12), patient quality of life was assessed. **There were statistically significant differences and clinically significant differences as determined by the MCID in overall patient quality of life.**   - For somatic function, patients in the intervention arm had a mean of 84.51 and a standard deviation of 11.25 while the patients in the control arm had a mean of 69.17 and a standard deviation of 15.56 (p < 0.001). - For emotional function, patients in the intervention arm had a mean of 86.69 and a standard deviation of 10.17 while the patients in the control arm had a mean of 68.91 and a standard deviation of 12.56 (p < 0.001). - For role function, patients in the intervention arm had a mean of 84.45 and a standard deviation of 11.51 while the patients in the control arm had a mean of 67.45 and a standard deviation of 13.34 (p < 0.001). - For social function, patients in the intervention arm had a mean of 83.45 and a standard deviation of 11.52 while the patients in the control arm had a mean of 69.08 and a standard deviation of 15.56 (p < 0.001). - For cognitive function, patients in the intervention arm had a mean of 84.64 and a standard deviation of 10.54 while the patients in the control arm had a mean of 66.98 and a standard deviation of 15.68 (p < 0.001). |
| Ji et al., 2022 | Understand the effect of a nurse-led education intervention on the patients' quality of life after thyroid surgery for a thyroid tumor.. | “Based on the control group, the experimental group adopted high-quality nursing: (1) A nursing team was established, and regular training and evaluation were conducted to improve the nursing skills, nursing awareness, and nurs-ing quality of nursing staff. (2) Disease-knowledge education was provided to improve patients’ awareness of their diseases and enhance the confidence in treatment. (3) Clinical Psychological evaluations of patients were conducted to formulate targeted psychological guidance programs according to the patient’s personality characteristics as patients are prone to negative emotions for surgery. Multiple psycholog-ical comfort and guidance were provided for patients with severe anxiety or depression to relieve the patients’ negative emotions; (4) preoperative nursing staff should comprehensively and systematically understand the patient’s drug allergy history and clinical data and guide the patient to relieve fear by adjusting breathing; (5) the patients’ vital signs and drainage conditions were closely observed during the operation to ensure tube patency and smooth breathing; (6) a clean and tidy treatment environment was provided for the patients after surgery, and the patients were given drugs regularly, with clinical reactions closely observed to avoid adverse events; (7) the patients were instructed by nursing staff to perform breathing and relaxation exercises, with relaxing music to divert their attention and reduce pain. Measures for severe pain were adopted if necessary. (8) Dietary instruction was provided according to the actual situation of the patients, including diets with nutritional balance, less raw and cold food, and food with more protein and vitamins; (9) reasonable rehabilitation training was provided, and patients were instructed to visit the hospital for review at regular intervals after discharge.” | Using the Hospital Anxiety and Depression Scales (HADS), patient quality of life was assessed. **There were statistically significant differences and clinically significant differences as determined by the MCID in overall patient quality of life.**   - For the HADS, patients in the intervention arm had a mean of 4.65 and a standard deviation of 1.02 while the patients in the control arm had a mean of 14.62 and a standard deviation of 1.96 (p < 0.001). |
| Zhao et al., 2021 | Understand the effect of a psychological nursing intervention program on the patients’ quality of life after surgery for lung cancer. | “Preoperative psychological care: (1) Preoperative assessment: the mental health status of young patients with lung cancer was evaluated such as their personality characteristics, cultural background,economic situation and social environment were further analyzed. The level of education and nursing ability of the family members were also assessed and explained the importance of psychological intervention to their family members and enable the family members to cooperate well with the patient’s psychological nursing intervention; (2) Prior operation guidance: explain the detail of the surgery can improve the condition of patients with lung cancer and provide a comprehensive introduction of the qualifications and professional skills to the medical staffs in the treatment area, so as to earn the trust of these patients to cooperate with treatment and care in a more positive manner; (3) Strengthen family and social support: several factors such as the risk of surgery, postoperative pain, and encouraged distaste may increase the psychological burden on young lung cancer patients, so patients could communicate with their families and friends who should give more comfort and encouragement to young patients. So that patients could fully feel the warmth of family and social support, so as to reduce the psychological pressure and promote them to face the operation with a more positive attitude.  Postoperative care: (1) Timely feedback: timely feedback to the patients using encouraging, positive words and tell the patients to cooperate with the treatment, so the tension of the body and mind may be relaxed; (2) Standardize pain management: strictly comply with thedoctor’s advice such as talk, listen to music and using painkillers properly; (3) Strong theory: after the operation, we should further strengthen the inspection of these young patients, make them clear the status of their mental health and give them psychological counseling and psychological comfort.Family members should encourage and comfort the patients, provide support and understanding for these patients, let the patients feel the care and loves of the family. Family members also need to communicate with the attending doctors and responsible nurses to under-stand the patient’s psychological dynamics and develop effective prevention strategies; (4) Discharge psychology refers to Guide: inform young lung cancer surgery patients that their post operative rehabilitation needs a process and a comprehensively enhance of the psychological tolerance and coping ability. Gradually they may accept the changes of body postoperative, then positively adjust their mindset to better match follow-up treatment, insist on lung rehabilitation function exercise and change their lifestyle.” | Using the self-rating anxiety scale (SAS) and self-rating depression scale (SDS), patient quality of life was assessed. **There were statistically significant differences and clinically significant differences as determined by the MCID in the SDS. There were statistically significant differences in SAS, but no MCID has been created yet.**   - For the SAS, patients in the intervention arm had a mean of 43.5 and a standard deviation of 6.2 while the patients in the control arm had a mean of 60.8 and a standard deviation of 7.7 (p < 0.01). - For the SDS, patients in the intervention arm had a mean of 48.5 and a standard deviation of 8.0 while the patients in the control arm had a mean of 57.8 and a standard deviation of 8.7 (p < 0.01). |
| Turkdogan et al., 2022 | Understand the effect of an educational video intervention on the patients’ quality of life after surgery for head or neck cancer. | “Patients in the intervention arm were given access to an educational platform called Precare. This platform is designed to provide individuals with a concise, animated video explaining the details of their diagnosis, how to adequately prepare for their upcoming surgery, what to expect during their hospital stay, and what steps they can take to help the recovery process at the hospital and on discharge home. To decrease the limitations of health literacy and language barriers, videos were created using a 6th-grade literacy level and were available in both official Canadian languages (English and French). Subtitles were created with professional translations in the most commonly spoken 20 languages in Canada. A multidisciplinary approach was taken, and the content was reviewed by participating otolaryngologists in our department, along with ancillary health care clinicians, including a nurse, nutritionist, speech language pa-thologist, respiratory therapist, physiotherapist, oncology-focused psychologist, and radiation oncologist. This allowed the videos on the platform to not only contain medical and sur-gical information concerning the diagnosis and treatment, but also expand on psychosocial elements, such as mental health and family life, in the recovery process.The oncology nurse assisted patients in first accessing theplatform, guiding them to the video that was specific to their surgery and providing them with written information on how to access the video for future reference if needed. To facilitate access for patients for whom navigating web-based platforms was more challenging, patients were additionally given the option to view the video in the clinic on an iPad (Apple) that was available for their use. Patients in the control arm did not gain access to the educational platform. Regardless of their study arm, all patients received standard in-person preoperative teaching by the health care staff and were given the opportunity to discuss any questions or concerns regarding their surgical care with their attending surgeon. All patients underwent surgery as per routine and had follow-up at 1 week and 1 month postoperatively.” | Using the European Organisation For Research and Treatment of Cancer Quality of Life Questionnaire (EORTC-QLQ), patient quality of life was assessed. **There were statistically significant differences in the EORTC QLQ. No MCID has been created yet.**   - For the amount of information received about accessible services, patients in the intervention arm had a mean of 196 while the patients in the control arm had a mean of 131 (Cohen d= 1.281). - For the information received about the treatment, patients in the intervention arm had a mean of 195 while the patients in the control arm had a mean of 143 (Cohen d= 1.05). - For medical tests, patients in the intervention arm had a mean of 129 while the patients in the control arm had a mean of 131 (Cohen d= 0.81). |
| Yu et al., 2022 | Understand the effect of a nurse-led telephone and Internet-based supportive care intervention on the patients’ quality of life after surgery for esophageal cancer. | “Before discharge: In addition to conventional care, the intervention group received nurse-led telephone and Internet-based supportive care. The supportive care team provided intervention. The nurses sent a pamphlet to each patient to introduce the supportive care plan. The nurses would make telephone calls to the patients regularly. The Internet-based supportive care was performed by a WeChat group. Patients joined in the group before discharge and asked questions anytime they needed, and the nurses regularly answered the questions online every day.  After discharge: The nurse called each patient for a one-on-one discussion after discharge once a week in the first two months, twice a month in months 3–4, and once a month in months 5–6. The follow-up telephone call focused on the patients' nutritional status, symptoms after esophagectomy, and psychological issues. The patients' eating situation would be evaluated using the simple diet self-assessment tool (SDSAT), which nurses taught patients to use before discharge. This tool also was available on a poster displayed on the wall of the ward (Figure 1). Nurses would provide dietary guidance based on the results of the SDSAT. The SDSAT scores ranged from 1 to 5, with higher scores meaning a better diet. Patients who scored 5 points did not need nutritional intervention. Patients who got 4 points would be advised to eat food rich in calories and protein and take nutritional supplements as needed. Patients who scored 3 points were instructed to take oral nutrition supplements high in calories and protein in addition to their normal diet. Patients who received 1 or 2 points were advised to go to the hospital's nutritional clinic for enteral or parenteral nutrition. These follow-up phone call took one afternoon per week. Nurses also asked about the patients' symptoms after surgery and answered their questions, for example, regarding pain, reflux, or cough. They recommended medical visits when necessary. The nurses asked patients if they had any psychological problems and provided counseling. Each of these telephone contacts lasted as long as the patient's desired, usually between 20 and 30 min. The nurses managed the WeChat group, using it to answer patients' questions. For instance, they organized and sent out answers to the most common 10 questions, information concerning common oral nutritional supplements (Figure 2), and articles related to postoperative rehabilitation and nutrition (Figure 3). The patients could upload pictures (Figure 4) and videos to the group and communicate with nurses and other patients. Different nurses were responsible for the WeChat group for a week inturn and answering questions at any time.” | Using the European Organisation For Research and Treatment of Cancer Quality of Life Questionnaire (EORTC-QLQ), patient quality of life was assessed. **There were statistically significant differences in the EORTC QLQ. No MCID has been created yet.**   - For total health/quality of life, patients in the intervention arm had a mean of 81.93 and a standard deviation of 12.58 while the patients in the control arm had a mean of 65.55 and a standard deviation of 16.76 (p = 0.000). |
| Yuan et al., 2022 | Understand the effect of a nurse-led follow-up intervention on the patients’ quality of life after hepatic artery interventional chemoembolization for hepatocellular carcinoma. | “Patients in the comprehensive group were given a comprehensive nursing modality for intervention, as follows:  Preoperative care: when patients were admitted,the hospital ward environment and work schedule were introduced to remove unfamiliarity and close the distance between nurses and patients; on-site lectures were provided to patients to alleviate their negative psychology such as fear,nervousness, and anxiety; and the benefits, principles, and priorities of interventional procedures were explained in detail. Explanation of successful treatment cases can enhance patients’ confidence in nursing care so that they can actively and effectively cooperate with nursing care with an optimistic attitude; creating a warm and comfortable hospital environment for patients, keeping the ward quiet, and not disturbing patients’ rest; in preoperative preparation,interventional devices and medications and iodine allergy tests were performed, patients fasted for 6 hours before surgery, protective medications were routinely given 30 minutes before surgery, blood pressure was monitored regularly, and blood pressure management was performed promptly in hypertensive patients.  Intraoperative care:during the intervention, medical and nursing staff com-municated more with patients to reduce their tension and avoid vasospasm, thereby guaranteeing the smooth performance of the interventive procedure and shortening the duration of the intervention. During the injection of contrast agent, medical staff closely monitored the patient’s reaction and checked for palpitations, shortness of breath, chest tightness, and other symptoms; if the patient had reactions such as nausea and vomiting when injecting chemotherapy drugs, they immediately cleaned up the vomit and turned patient's head and neck sideways to avoid accidental aspiration of the vomit.  Postoperative nursing: patients should rest in bed for 24 hours after surgery, in a supine position, with sandbag compression at the injection site for 6 hours and limbs braked in extension at the injection site for 6 hours to avoid bending and pressure; patients should be helped to move their bodies slightly to avoid prolonged local pressure to increase their comfort; patients will have fever after interventional treatment,which usually lasts for about a week. Generally speaking, no special treatment is needed for body temperature below 38.5°C, which can be reduced by rubbing with warm water; if the fever is higher than 38.5°C,diclofenac sodium 1/2 anal plug or physical cooling can be given. Due to the side effects of chemotherapy, patients may experience nausea, vomiting, and loss of appetite aftertreatment, so the gastrointestinal tract should be well cared for; patients can resume a normal diet after 3 days, with attention to eating more fresh vegetables, fruits with high vitamin content, and high-calorie foods, appropriately limiting the intake of high-fat foods, and drinking more water to promote the excretion of the contrast agent; keeping the bowels open and advising patients to avoid violent coughing and straining to defecate to prevent bleeding. Afterliver cancer intervention, swelling and pain at the liver site may occur to varying degrees, usually within 1-2 hours after the procedure, but will gradually ease within 35 days. If it does not remit, the cause should be promptly explained and the site, nature, degree, and duration of abdominal pain should be closely monitored and alerted; for ruptured he-patocellular carcinoma bleeding, equivalent treatment should be given to alleviate the patient’s concern; patients with pain can be given appropriate relief measures, including distraction and instruction in relaxation techniques for mild pain, intravenous flurbiprofen for moderate pain, and in-tramuscular pethidine hydrochloride for severe pain.  Health guidance: after surgery, patients should be actively communicated with and guided to establish confidence in overcoming the disease; scientific daily life and medication plans should be formulated, and family members should be instructed to urge patients to correct bad habits; diet plans should be formulated for patients according to their dietary habits, and they should be encouraged to eat more food with high calorie, vitamin, and protein content, as these will meet nutritional needs and improve resilience; communication with family members, explaining to them the role of family members in care, allowing them to participate in treatment to relieve patients’ mental burden, etc.” | Using the Quality of Life Rating Scale (SF-36), the Numeric Rating Scales (NRS), the Self-Rating Anxiety Scale (SAS), and the Self-Rating Depression Scale (SDS), patient quality of life was assessed. **There were statistically significant differences in all metrics. There were clinically significant differences as determined by the MCID in the SF-36 and the SDS. No MCID has been created yet for the NRS or SAS.**   - For the Numeric Rating Scales (NRS), patients in the intervention arm had a mean of 2.99 and a standard deviation of 0.97 while the patients in the control arm had a mean of 4.85 and a standard deviation of 1.11 (p < 0.05). - For the Self-Rating Anxiety Scale (SAS), patients in the intervention arm had a mean of 42.17 and a standard deviation of 2.65 while the patients in the control arm had a mean of 50.48 and a standard deviation of 3.14 (p < 0.001). - For the Self-Rating Depression Scale (SDS), patients in the intervention arm had a mean of 40.08 and a standard deviation of 2.19 while the patients in the control arm had a mean of 52.17 and a standard deviation of 3.26 (p < 0.001). |

**Appendix S5:** Patient-Reported Quality Outcomes Reported

| Patient-Reported Quality Outcome | Studies | Description | Range | Minimal clinically important differences (MCID) |
| --- | --- | --- | --- | --- |
| Quality of Life Scales (QLQ-c30)^7–9^ | Malmstrom et al., 2016  Koet et al., 2021  Verschuur et al., 2009  Mertz et al., 2017  Sussman et al., 2017  Li et al., 2016  Temur and Kapucu, 2019  Ross et al., 2005  Quist et al., 2018  Bjerring et al., 2020  Sui et al., 2020  Turkdogan et al., 2022  Yu et al., 2022 | Questionnaire with multi-item scales and single items measuring cancer patient’s physical, psychological, and social functions. | 0-100 | The MCID for improvement and deterioration, respectively were: physical (6, 9), role (14, 12), and cognitive functioning (8, 8); global health status (7, 4), fatigue (12, 9), and motor dysfunction (4, 5).^10^ |
| Hospital Anxiety and Depression Scales (HADS)^11^ | Koinberg et al., 2004  Mertz et al., 2017  Sui et al., 2020  Ross et al., 2005  Ji et al., 2022 | 2-5 minute questionnaire to quantify the anxiety and depression of a patient in the hospital. There are 7 questions to assess anxiety and 7 questions for depression. | Each question is scored from 0-3. Each category (either anxiety or depression) can have a range from 0-21. | A 1.7 point shift, either in the positive or negative direction, can be considered clinically significant.^12^ |
| Symptom Distress Scale (SDS)^13–15^ | McCorkle et al., 2009  Wallen et al., 2012  Zheng and Jiang, 2019  Zhao et al., 2021  Yuan et al., 2022 | Questionnaire with 13 different symptoms that could be experienced by the patient. A score of 1-5 is given for each symptom and the sum of all 13 symptoms is used to calculate the final score. | 13-65 | A four point shift, either in the positive or negative direction, can be considered clinically significant.^16^ |
| 12-Item Short Form Health Survey (SF-12)^17,18^ | McCorkle et al., 2009  Hu et al., 2022  Zheng and Jiang, 2019 (SF-36)  Yuan et al., 2022 (SF-36) | 12 item questionnaire that measures physical and mental health. | 0-100 | A 5-8 point shift, either in the positive or negative direction, can be considered clinically significant.^19^ |
| Functional Assessment of Cancer Therapy (FACT-C)^20^ | Harrison et al., 2011  Young et al., 2013  Ding et al., 2022 | Questionnaire that includes four general sections for health related quality of life and one section for colorectal cancer specifically. | 0-100 | A 5-8 point shift, either in the positive or negative direction, can be considered clinically significant.^21^ |
| Functional Assessment of Cancer Therapy (FACT-B)^22^ | Zhou et al., 2020 | Questionnaire with 37 items, grouped into 5 subscales, that are measured using the 5 point Likert scale. | 0-100 | A 7-8 point shift, either in the positive or negative direction, can be considered clinically significant.^23^ |
| Self-rating Anxiety  Scale (SAS)^24^ | Zhu et al., 2021  Zhao et al., 2021  Yuan et al., 2022 | Questionnaire with 20 questions, scored from 1-4 points to measure the patient’s current level of anxiety. | 20-80 | No MCID available yet.^25^ |
| Self-rating Depression Scale (SDS)^26^ | Zhu et al., 2021 | Questionnaire with 20 questions, scored from 1-4 points to measure the patient’s current level of depression. | 20-80 | A 4.9 point shift, either in the positive or negative direction, can be considered clinically significant.^27^ |
| Profile of Mood States^28^ | Watson et al., 1988  Allard 2007  Boesen et al., 2005 | Questionnaire with 65 items, scored with the Likert-scale rated, to assess a variety of mood states. | 7-35 (7 items in each subscale). | A 5.6 point shift, either in the positive or negative direction, can be considered clinically significant.^29^ |
| Gracely Pain Scale^30^ | Wallen et al., 2012 | Visual analog scale to measure the patient’s self-reported pain. | 0-20 | No MCID available yet. |
| Center  for Epidemiologic Studies Depression Scale^31^ | Wallen et al., 2012 | Questionnaire with 20 items to assess a patient’s level of depression. | 0-60 | A 11 point shift, either in the positive or negative direction, can be considered clinically significant.^32^ |
| Stoma Self-Efficacy Scale^33^ | Shujuan et al., 2018 | Questionnaire with 29 items, which were answered with categorical descriptions (not being confident at all, slightly confident, fairly confident, highly confident, and extremely confident). | 29-145 | No MCID available yet. |
| Numerical Rating Scale for pain intensity^34^ | Francke et al., 1997  Yuan et al., 2022 | Visual analogue and graphic rating scales to assess a patient’s pain. | No given range as some questions are scored from 1-5 while others have categorical descriptions (severe, moderate, low pain). | No MCID available yet. |
| American Pain Society- patient questionnaire^35^ | Bahrami et al., 2012 | Questionnaire with 12 questions to better understand the patient’s pain. | 0-100 | No MCID available yet. |
| Mood Adjective Check List (MACL)^36^ | Ohlsson-Nevo et al., 2017 | Questionnaire with 71 items that describe different moods. The patient rates how well the mood describes their current state. | 71-284 | No MCID available yet.^37^ |

**References:**

1. Yefimova, M. *et al.* Palliative Care and End-of-Life Outcomes Following High-risk Surgery. *JAMA Surg* **155**, 138 (2020).
2. Lamont, E. B. A Demographic and Prognostic Approach to Defining the End of Life. *Journal of Palliative Medicine* **8**, s-12-s-21 (2005).
3. Hui, D. *et al.* Concepts and Definitions for “Actively Dying,” “End of Life,” “Terminally Ill,” “Terminal Care,” and “Transition of Care”: A Systematic Review. *Journal of Pain and Symptom Management* **47**, 77–89 (2014).
4. Wang, S.-Y. *et al.* End-of-Life Care Intensity and Hospice Use: A Regional-level Analysis. *Medical Care* **54**, 672–678 (2016).
5. Barrett, N. & Wright, M. E. Key Elements of Advanced Practice Provider Integration. *The Journal for Nurse Practitioners* **15**, 370-373.e2 (2019).
6. Vetter, T. R. & Mascha, E. J. Defining the Primary Outcomes and Justifying Secondary Outcomes of a Study: Usually, the Fewer, the Better. *Anesthesia & Analgesia* **125**, 678–681 (2017).
7. Kaasa, S. *et al.* The EORTC Core Quality of Life questionnaire (QLQ-C30): validity and reliability when analysed with patients treated with palliative radiotherapy. *European Journal of Cancer* **31**, 2260–2263 (1995).
8. Fayers, P. & Bottomley, A. Quality of life research within the EORTC—the EORTC QLQ-C30. *European Journal of Cancer* **38**, 125–133 (2002).
9. Bergman, B., Aaronson, N. K., Ahmedzai, S., Kaasa, S. & Sullivan, M. The EORTC QLQ-LC13: a modular supplement to the EORTC core quality of life questionnaire (QLQ-C30) for use in lung cancer clinical trials. *European Journal of Cancer* **30**, 635–642 (1994).
10. Maringwa, J. *et al.* Minimal clinically meaningful differences for the EORTC QLQ-C30 and EORTC QLQ-BN20 scales in brain cancer patients. *Annals of Oncology* **22**, 2107–2112 (2011).
11. Stern, A. F. The Hospital Anxiety and Depression Scale. *Occupational Medicine* **64**, 393–394 (2014).
12. Lemay, K. R., Tulloch, H. E., Pipe, A. L. & Reed, J. L. Establishing the Minimal Clinically Important Difference for the Hospital Anxiety and Depression Scale in Patients With Cardiovascular Disease. *Journal of Cardiopulmonary Rehabilitation and Prevention* **39**, E6–E11 (2019).
13. McCorkle, R. & Young, K. Development of a symptom distress scale. *Cancer Nurs* **1**, 373–378 (1978).
14. McCorkle, R. The measurement of symptom distress. *Seminars in Oncology Nursing* **3**, 248–256 (1987).
15. McCorkle, R. & Quint-Benoliel, J. Symptom distress, current concerns and mood disturbance after diagnosis of life-threatening disease. *Social Science & Medicine* **17**, 431–438 (1983).
16. Sloan, J., Symonds, T., Vargas-Chanes, D. & Fridley, B. Practical Guidelines for Assessing the Clinical Significance of Health-Related Quality of Life Changes within Clinical Trials. *Drug Information J* **37**, 23–31 (2003).
17. Ware, J. E., Kosinski, M. & Keller, S. D. A 12-Item Short-Form Health Survey: Construction of Scales and Preliminary Tests of Reliability and Validity. *Medical Care* **34**, 220–233 (1996).
18. Jenkinson, C. *et al.* A shorter form health survey: can the SF-12 replicate results from the SF-36 in longitudinal studies? *Journal of Public Health* **19**, 179–186 (1997).
19. Jacquet, C. *et al.* Evaluation of the “Minimal Clinically Important Difference” (MCID) of the KOOS, KSS and SF-12 scores after open-wedge high tibial osteotomy. *Knee Surg Sports Traumatol Arthrosc* **29**, 820–826 (2021).
20. Ward, W. L. *et al.* Reliability and validity of the Functional Assessment of Cancer Therapy-Colorectal (FACT-C) quality of life instrument. *Quality of Life Research* **8**, 181–195 (1999).
21. Yost, K. *et al.* Minimally important differences were estimated for the Functional Assessment of Cancer Therapy–Colorectal (FACT-C) instrument using a combination of distribution- and anchor-based approaches. *Journal of Clinical Epidemiology* **58**, 1241–1251 (2005).
22. Hahn, E. A., Segawa, E., Kaiser, K., Cella, D. & Smith, B. D. Validation of the Functional Assessment of Cancer Therapy-Breast (FACT-B) quality of life instrument. *JCO* **33**, e17753–e17753 (2015).
23. Jayadevappa, R., Cook, R. & Chhatre, S. Minimal important difference to infer changes in health-related quality of life—a systematic review. *Journal of Clinical Epidemiology* **89**, 188–198 (2017).
24. Zung, W. W. K. A Rating Instrument For Anxiety Disorders. *Psychosomatics* **12**, 371–379 (1971).
25. Celik, Y., Thunström, E., Strollo, P. J. & Peker, Y. Continuous positive airway pressure treatment and anxiety in adults with coronary artery disease and nonsleepy obstructive sleep apnea in the RICCADSA trial. *Sleep Medicine* **77**, 96–103 (2021).
26. Zung, W. W. K. A Self-Rating Depression Scale. *Arch Gen Psychiatry* **12**, 63 (1965).
27. Parker, S. L. *et al.* Minimum clinically important difference in pain, disability, and quality of life after neural decompression and fusion for same-level recurrent lumbar stenosis: understanding clinical versus statistical significance: Clinical article. *SPI* **16**, 471–478 (2012).
28. Pollock, V., Cho, D. W., Reker, D. & Volavka, J. Profile of Mood States: The Factors and Their Physiological Correlates: *The Journal of Nervous and Mental Disease* **167**, 612–614 (1979).
29. Schwartz, A. *et al.* Measurement of fatiguedetermining minimally important clinical differences. *Journal of Clinical Epidemiology* **55**, 239–244 (2002).
30. Gracely, R. H. Evaluation of multi-dimensional pain scales. *Pain* **48**, 297–300 (1992).
31. Eaton, W. W., Smith, C., Ybarra, M., Muntaner, C., & Tien, A. (2004). Center for Epidemiologic Studies Depression Scale: Review and Revision (CESD and CESD-R). In M. E. Maruish (Ed.), The use of psychological testing for treatment planning and outcomes assessment: Instruments for adults (pp. 363–377). Lawrence Erlbaum Associates Publishers.
32. Haase, I., Winkeler, M. & Imgart, H. Ascertaining minimal clinically meaningful changes in symptoms of depression rated by the 15‐item Centre for Epidemiologic Studies Depression Scale. *Evaluation Clinical Practice* **28**, 500–506 (2022).
33. Bekkers, M. J. T. M. PhD; Van Knippenberg, F. C. E. PhD; Van Den Borne, H. W. PhD; Van Berge-Henegouwen, G. P. PhD Prospective Evaluation of Psychosocial Adaption to Stoma Surgery, Psychosomatic Medicine: March/April 1996 - Volume 58 - Issue 2 - p 183-191.
34. Scott, J. & Huskisson, E. C. Graphic representation of pain: *Pain* **2**, 175–184 (1976).
35. Ward, S. E. & Gordon, D. Application of the American Pain Society quality assurance standards. *Pain* **56**, 299–306 (1994).
36. Svensson, E. Response format and factor structure in mood adjective check lists. *Scand J Psychol* **18**, 71–78 (1977).
37. Kruis, A. L. *et al.* Integrated disease management interventions for patients with chronic obstructive pulmonary disease. *Cochrane Database of Systematic Reviews* (2013) doi:10.1002/14651858.CD009437.pub2.
