# Supplementary material for: Interdisciplinary interventions that improve patient-reported outcomes in perioperative cancer care: A systematic review of randomized control trials
Source: PLoS One. 2023 Nov 20;18(11):e0294599. doi: 10.1371/journal.pone.0294599 (PMC10659207; doi:10.1371/journal.pone.0294599)
Supplement: Attachment — Submitted filename: Review comments for manuscript number PONE-D-23-16022.docx [file pone.0294599.s003.docx]

**Title:** Interdisciplinary Interventions that Improve Patient-Reported Outcomes in Perioperative Cancer Care: A Systematic Review of Randomized Control Trials

**Version:** 1 **Date:** 24 July, 2023

**Reviewer’s report:**

The authors aim to characterize the structural and procedural components of interdisciplinary interventions in perioperative cancer care that have a positive impact in patient-reported outcomes. There is variability in the intervention used and interdisciplinary team members in interventional studies in perioperative cancer care showing improvement in patient-reported outcomes.

Overall, the study would seem to have constructive influence in practice as well as research.

As of my awareness following revisions should be made to the manuscripts.

- The term interdisciplinary intervention should be defined in the manuscript since there is a very thin line between definition of terms multidisciplinary and interdisciplinary in health research. Authors can go through a review entitled “Multidisciplinarity, interdisciplinarity and transdisciplinarity in health research, services, education and policy: 1. Definitions, objectives, and evidence of effectiveness” by Choi and Pak. This further seems necessary due to variability in the team processes in the included studies.
- Page no. 2: Abstract, Results, first sentence: patient-reported outcomes is appropriate in place of patient-reported quality outcomes.
- Page no. 4: The statement “Since the perioperative period of cancer care has an increased incidence of morbidity and mortality, it serves as an opportunity for leveraging diverse clinical team members to improve outcomes for patients” does not seem to be well supported by the cited literature. It would be better if the sentence is re-written or if appropriate literature supporting the statement be cited.
- Page no. 4: last paragraph, first sentence: systematic review would be appropriate be than narrative systematic review.
- Please go through manuscript again for eliminating typos, spelling, grammar, and phrasing errors.
