# Supplementary material for: Interdisciplinary interventions that improve patient-reported outcomes in perioperative cancer care: A systematic review of randomized control trials
Source: PLoS One. 2023 Nov 20;18(11):e0294599. doi: 10.1371/journal.pone.0294599 (PMC10659207; doi:10.1371/journal.pone.0294599)
Supplement: Attachment — Submitted filename: Review Comments.docx [file pone.0294599.s004.docx]

Dear Authors,

Your article “**Interdisciplinary Interventions that Improve Patient-Reported Outcomes in Perioperative Cancer Care: A Systematic Review of Randomized Control Trials**” systematic review of randomized meets the criteria and requirement of PLOS One and delivers some notable information’s and context.

I find your writing mostly to be clear and concise and appropriately deliver the theme /importance of team while managing perioperative cancer patients. The paper properly highlights the importance of surgery, its complications and the interdisciplinary interventions such as education, engaging with patient caregivers, communication, longitudinal follow-up in perioperative period in cancer patients for better patient reported outcomes. I found the search strategy, PRISMA-P flow charts and all the relevant information attached within and found the article genuinely suitable for publication in this journal. However, I do have some minor comments regarding your paper. Please try to address these comments and suggestions.

1. Page 3 Para 2^nd^:

“Both cancer and its treatment touches and often impairs diverse aspects of quality of life (e.g., physical, emotional, existential, and social); because needs fluctuate over time, an interdisciplinary team can be leveraged to support patients’ changing needs so that the care is customized to each patient’s changing needs.”

**Comments:** The theme I got from this sentence is that “Cancer and its treatment impairs the quality of life of patients (physical, emotional, existential, and social) and hence interdisciplinary support and team can be established in order to support the patients’ changing needs.” However, this sentence while reading sounds a bit complex and mismatched and can be complex for the readers. Hence, I request you to please re-write the sentence without destroying the essence.

2. Page 7, Paragraph 2nd:

“The main outcomes collected were any form of patient-reported outcomes (PROs).” In the same paragraph where you’ve also written “Through different patient-reported outcomes……”. This sentence also lacks PROs abbreviation consistency.

**Comments:** You have already abbreviated patient-reported outcomes as PROs in Page 4, 4^th^ Paragraph. Please maintain abbreviation consistency.

3. Page 12-13: “There are not enough included studies, and not enough information regarding team structures in the included studies to draw conclusions about the most effective team structures in improving PROs in perioperative cancer care.”

**Comments:** You mentioned how there are not enough studies and information regarding team structures to draw conclusion regarding PROs in perioperative cancer care. Regarding this statement you haven’t addressed the various reasons for such low number of studies in the discussion section. I feel this needs to be addressed since you have mentioned your aim to identify the team structure responsible for interdisciplinary interventions for better PROs.

4. Since you’ve mentioned the aim of the study to identify the team structure and process while exhibiting interdisciplinary interventions. Your conclusion includes the interventions that were responsible for improved PROs. However, it does not give the idea on the non-physician intervention clinical team member such as advanced practice providers (APP), including nurse practitioners and physician assistants, clinical nurse specialists, and registered nurses. Do any of these needs to be included in the conclusion section? Since you’ve mentioned how advanced practice providers in the intervention, regardless of the specific role they performed were associated with an improvement in PROs compared to the control group and the roles of nurse practitioners in cancer patients. I feel your conclusion can be more concrete if you mention both the idea of the non-physician clinical team members responsible and the interventions provided for better PROs. Regarding the limited papers of the inclusion of APP, you can go ahead with the similar information in your conclusion. You can also include the role of communication as an intervention in the conclusion section since you have addressed how communication has helped in improving PROs as an interventional role (Page 13, para 2^nd^)
5. Please look out for the minor typing errors, mismatched spelling and punctuation marks.

Thank you. I feel these comments are very simple and can be easily addressed. Best of luck!
