# Supplementary material for: Interdisciplinary interventions that improve patient-reported outcomes in perioperative cancer care: A systematic review of randomized control trials
Source: PLoS One. 2023 Nov 20;18(11):e0294599. doi: 10.1371/journal.pone.0294599 (PMC10659207; doi:10.1371/journal.pone.0294599)
Supplement: Attachment — Submitted filename: Perioperative SR Response to Reviewers.docx [file pone.0294599.s006.docx]

Additional Editor Comments:

The authors have addressed the majority of the reviewers' comments. To facilitate the manuscript's acceptance, I recommend addressing some of the remaining minor comments.

- Thank you for taking the time to review our manuscript. We have addressed the remaining minor comments below.

Introduction:

1. Specify research objectives and gaps in the literature.
   a. We have made the appropriate changes in the manuscript:
      i. The gaps in literature are addressed in this sentence: “Despite a recent surge in interventions integrating interdisciplinary teams in perioperative cancer care, individual components of interventions, such as specific structures and processes have not been analyzed.”
      ii. The research objectives are addressed: “The purpose of this paper was to identify the structures (who is on the team and what are their roles) and processes (how the team functions and communicates) in existing interdisciplinary interventions that improved perioperative patient reported outcomes (PROs) in randomized controlled trials to garner insights on how future interventions might improve PROs in perioperative cancer care. We also aimed to understand the various components of interventions and the impact they had on PROs. This includes the types of interventions, specific team member roles, and caregiver involvement.”
2. Provide citations to support claims.
   a. All claims have been double checked to ensure they are supported with citations.
3. Improve transition and flow between sections.
   a. The last sentence of the first paragraph was updated to better tie into the second paragraph about quality of life for cancer care: “Leveraging non-physician clinical team members (e.g. advanced practice providers, clinical nurse specialists, and registered nurses) to participate on interdisciplinary teams can improve the quality of life for patients, especially in the context of cancer care.”
   b. The first sentence of the third paragraph builds on the second paragraph by specifically providing one time point (the perioperative period) where teams can play a role in improving patient care: “The perioperative period is an exemplary context in which teams may play an important role in fostering better outcomes in cancer care.”
   c. The last sentence of the fourth paragraph helps set up the gap in literature, which leads into the fifth paragraph: “The perioperative period is an exemplary context in which teams may play an important role in fostering better outcomes in cancer care.”
4. Offer more specific examples during discussions.
   a. We have included specific examples within the Introduction to better support the points made: “ For example, in a randomized control trial where a nurse practitioner joined the oncology team to discuss hospice, living wills, and advanced directives with patients metastatic cancer, there was an improvement in the patients’ quality of life, overall physical, mental, and emotional wellbeing.”
5. Support statistics with references.
   a. All statistics have been double checked to ensure they are supported with citations.
6. Condense background information.
   a. We have removed unnecessary information in the Introduction where appropriate to condense this section.
7. Clarify terminology.
   a. We have clarified all the terms used within the Introduction:
      i. “A team can be either bounded, where members have static, defined roles, or dynamic, where members adjust roles continuously based on the task.”
      ii. “Leveraging non-physician clinical team members (e.g. advanced practice providers, clinical nurse specialists, and registered nurses) to participate on interdisciplinary teams can improve the quality of life for patients, especially in the context of cancer care”
      iii. “During the perioperative period, surgeons and surgical teams have many complex tasks which require interdisciplinary collaboration: (1) communicate the purpose and expect results of surgery (which may be curative, life-extending, or palliative), (2) identify patients who are appropriate for surgery based on relative risk of frailty or life expectancy, (3) optimize patients for surgery to minimize risks, and (4) quickly identify and manage complications postoperatively.”
8. Explicitly connect the systematic review to the introduction.
   a. We have adjusted the Introduction’s last paragraph to explicitly connect the systematic review to all the points made in the introduction:
      i. “The purpose of this paper was to identify the structures (who is on the team and what are their roles) and processes (how the team functions and communicates) in existing interdisciplinary interventions that improved perioperative patient reported outcomes (PROs) among patients with cancer in randomized controlled trials to garner insights on how future interventions might improve PROs in perioperative cancer care.”
9. State hypotheses or research questions.
   a. We have included the research questions in the Introduction:
      i. “The purpose of this paper was to identify the structures (who is on the team and what are their roles) and processes (how the team functions and communicates) in existing interdisciplinary interventions that improved perioperative patient reported outcomes (PROs) in randomized controlled trials to garner insights on how future interventions might improve PROs in perioperative cancer care. We also aimed to understand the various components of interventions and the impact they had on PROs to guide future interventions directed towards improving PROs in perioperative cancer care. This includes the types of interventions, specific team member roles, and caregiver involvement.”
10. Emphasize the significance of the systematic review.
    a. We have emphasized the significance of the systematic review within the Introduction:
       i. “Through this systematic review, we sought to understand how clinical team members might improve perioperative care and how their roles might be further modified or extended to improve perioperative care quality”
       ii. “The purpose of this paper was to identify the structures (who is on the team and what are their roles) and processes (how the team functions and communicates) in existing interdisciplinary interventions that improved perioperative patient reported outcomes (PROs) among patients with cancer in randomized controlled trials to garner insights on how future interventions might improve PROs in perioperative cancer care.”

Methods:

11. Structure the section with subsections.
    a. We have included the following subsections within the Methods:
       i. Study Selection, Data Collection, and Data Synthesis
12. Summarize key components of the search strategy.
    a. The key components utilized in the search strategy, as well as the entire search strategy itself, are included in the Methods:
       i. “Key concepts included in the search strategy included randomized control trials, cancer care, and clinical team members as shown in Appendix S1.”
13. Provide rationales for inclusion and exclusion criteria.
    a. We have included a detailed explanation of why certain inclusion/ exclusion criteria were included:
       i. “We also excluded studies of art/music therapy, exercise therapy, diet changes, or spiritual therapy for patients because these interventions have been extensively studied in the literature as effective programs on their own. These represent specific clinical interventions that have been shown to be effective on their own, regardless of what team members facilitating the intervention, and we wanted this systematic review to focus on the components of interventions (e.g. team structure and processes) that impacted PROs.”
       ii. Further rationales are also included in Table 1
14. Explain the rationale behind excluding certain interventions.
    a. Specific interventions such as art/music therapy, exercise therapy, diet changes, or spiritual therapy were excluded in this study. Detailed rationale behind these exclusions is provided in the Methods:
       i. ““We also excluded studies of art/music therapy, exercise therapy, diet changes, or spiritual therapy for patients because these interventions have been extensively studied in the literature as effective programs on their own. These represent specific clinical interventions that have been shown to be effective on their own, regardless of what team members facilitating the intervention, and we wanted this systematic review to focus on the components of interventions (e.g. team structure and processes) that impacted PROs.”
15. Mention inter-rater agreement during data collection.
    a. We have included the numeric inter-rater reliability for each stage of screening:
       i. “During the title/abstract screening (inter-rater reliability; Cohen’s Kappa = 0.94022), we resolved all conflicts by a group consensus or by a “gold standard” reviewer (KG). We screened the full texts that remained after the title/abstract screening in a similar way. During the full-text screening (inter-rater reliability; Cohen’s Kappa = 0.84632), the reason for exclusion was also identified.”
16. Clarify criteria for determining "clinically meaningful" differences.
    a. We have defined MCID and have also included Appendix S5 that helps determines “clinically meaningful” differences:
       i. “MCID is defined as the smallest possible change in a reported outcome that has a clinical impact.^29,32^”
17. Define and distinguish "teaming" from traditional teamwork.
    a. We have defined “teaming” and distinguished it from traditional teamwork in the first paragraph of the Introduction.
       i. “A team can be either bounded, where members have static, defined roles, or dynamic, where members adjust roles continuously based on the task.^2,3^”
       ii. “The dynamic model of teaming is important in healthcare due to the dynamic nature of patient disease trajectories and medical care generally, allowing different members of the healthcare team to adjust their roles as needed to best take care of the patient.^4,5^”
18. Explain the significance of family involvement in decision-making.
    a. We have included the following sentence in the Results:
       i. “Caregiver involvement is pertinent because it serves as a way to define quality of care since in the case of serious illness or palliative populations, the patient themselves may have decreased decision making capacity and engagement of the family or caregiver ensures the patient's wishes are respected.”
19. Ensure accessibility and citation of appendices and supplementary materials.
    a. We have ensured the accessibility and citation of appendices and supplementary materials.

Discussion:

20. Discuss quality assessment criteria or tools.
    a. We have discussed and defined the quality assessment tool used:
       i. “The Cochrane Risk of Bias tool is an accepted risk of bias assessment for RCTs and considered seven domains: sequence generation, allocation concealment. blinding of participants and personnel, blinding of outcome assessors, incomplete outcome data, selective outcome reporting, and other sources of bias.”
21. Analyze potential limitations and biases in included studies.
    a. We have included potential limitations and biases of the included studies:
       i. “ The included studies were heterogeneous in nature, which did not allow for statistical analysis, thus limiting the conclusions formed in this study. Finally, the included studies did not provide sufficient information in the manuscript regarding the purpose of surgeon interaction with the patient during the perioperative period (communicate the purpose and expect results of surgery, identify patients who are appropriate for surgery, optimize patients for surgery to minimize risks, or identify and manage complications postoperatively), which may have added an additional layer of understanding.”
       ii. The included articles had no overall high risk of bias as per the Cochrane Risk of Bias tool.
22. Provide specific examples or statistics from reviewed studies.
    a. We have included specific examples from the included studies:
       i. “In the group setting, outcomes in the QLQ-c-30, Profile of Mood States, and MACL were improved, which may be attributed to patients being in the physical presence of each other and providing reassurance and camaraderie, accumulating knowledge together, and asking questions.^40,44,48,60,61,62,65,66,77^ For example, Koet et al., 2021 demonstrated a statistically significant difference in the QLQ-C30 at the one month follow up between the intervention arm (mean = 72.1) and the control arm (mean = 63.9).^44^”
       ii. “ In almost all the included perioperative studies, longitudinal patient contact was seen, but was especially exemplified by Mertz et al., 2017 where there were no improvements in patient reported outcomes at six months, but there were clinically and statistically significant improvements in the intervention group at twelve months.^46^”
23. Include a section on clinical implications of the findings.
    a. We have included clinical implications through the discussion as follows:
       i. “Due to limited resources and time for surgeons, clinical team members can take on an expanded role in cancer care to conduct patient/caregiver education or clinical follow-up with the patients.”
       ii. “ Given the well-known workforce constraints for palliative care in cancer, advanced practice providers are an ideal alternative to maintain quality of care while considering overall healthcare expenditure.83 Oncology advanced practice providers can lead follow-up visits and educational sessions for patients in addition to administrative and clinical responsibilities, making this a viable opportunity specifically in the context of perioperative cancer care.”
       iii. “Longitudinal follow-up and effective communication among the healthcare team, with referrals to healthcare team members, may be used to improve the quality of life for patients with cancer in the perioperative period.”
       iv. “Including caregivers in cancer interventions has also translated to improved patient satisfaction, suggesting it may be a worthwhile component to include in the treatment of patients with cancer in the perioperative period.”

Conclusion:

24. Specify practical applications and expected benefits of the intervention.
    a. We have included the following statement within the conclusion:
       i. “The intervention components found in this study can be leveraged in efforts to improve perioperative cancer care based on the resources available and the specific needs of the patients.”
25. Elaborate on the importance of factors like surgeon type and purpose of interaction.
    a. We have included the following statement within the conclusion:
       i. “In addition, different types of surgeons have different practices, workflows, and practice cultures. Interventions that work for one practice culture may need to be adapted to translate into other practice cultures.”
26. Summarize limitations of the systematic review for transparency.
    a. We have included limitations of the systematic review for transparency:
       i. “Our study can be considered in light of the following limitations. The incorporation of advanced practice providers in the interdisciplinary healthcare team is recent and only three articles that had an intervention with advanced practice providers were included from a limited body of literature. We anticipate an increasing number of publications with interventions that include advanced practice providers. The patient reported outcomes included were heterogeneous and thus a meta-analysis or further synthesis is not possible. Further, PROs hold the bias that patients may have their own individual reference points for answering the questions.^95^ Our abstraction form did not capture the type of surgeon, which may be relevant in understanding PROs in different perioperative contexts. Furthermore, the search strategy itself, and the screening process, may have missed certain articles. The included studies were heterogeneous in nature, which did not allow for statistical analysis, thus limiting the conclusions formed in this study. Finally, the included studies did not provide sufficient information in the manuscript regarding the purpose of surgeon interaction with the patient during the perioperative period (communicate the purpose and expect results of surgery, identify patients who are appropriate for surgery, optimize patients for surgery to minimize risks, or identify and manage complications postoperatively), which may have added an additional layer of understanding.”
